# Absorption of Phosphonium Cations and Dications into a Hydrated POPC Phospholipid Bilayer: A Computational Study

**DOI:** 10.1021/acs.jpcb.2c02212

**Published:** 2022-06-06

**Authors:** V. V. S. Pillai, P. Kumari, A. Benedetto, D. Gobbo, P. Ballone

**Affiliations:** † School of Physics, University College Dublin, Dublin 4, Ireland; ‡ Conway Institute for Biomolecular and Biomedical Research, University College Dublin, Dublin 4, Ireland; § Department of Sciences, University of Roma Tre, I-00154 Rome, Italy; ∥ School of Pharmaceutical Sciences and ISPSO, University of Geneva, Rue Michel-Servet 1, CH-1211, Geneva 4, Switzerland; ⊥ Computational and Chemical Biology, Fondazione Istituto Italiano di Tecnologia, I-16163 Genova, Italy

## Abstract

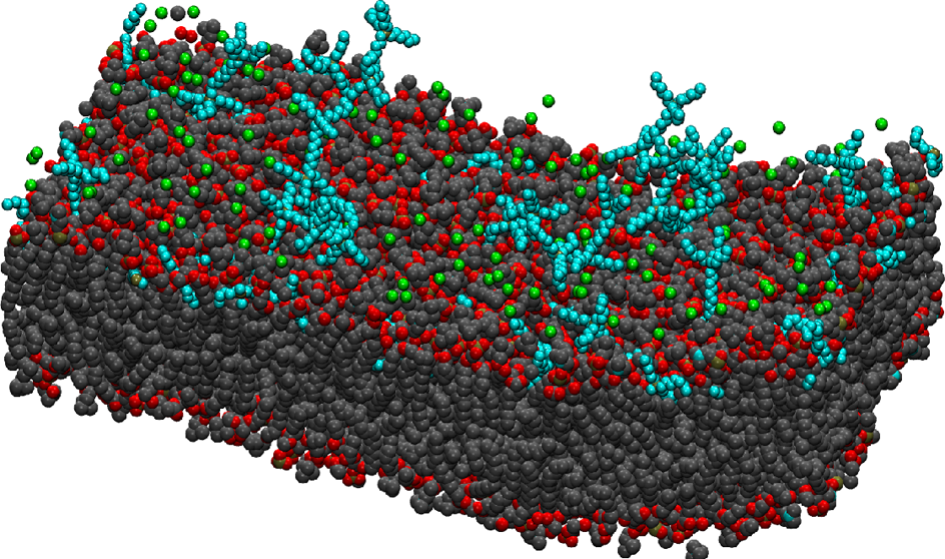

Molecular dynamics (MD) based on an empirical force field is applied to investigate the effect of phosphonium cations ([P_6,6,6,6_]^+^) and geminal dications ([DxC10]^2+^) inserted at *T* = 300 K into the hydration layer separating planar POPC phospholipid bilayers. Up to high concentration, nearly every added cation and dication becomes absorbed into the lipid phase. Absorption takes place during several microseconds and is virtually irreversible. The neutralizing counterions ([Cl]^−^, in the present simulation) remain dissolved in water, giving origin to the charge separation and the strong electrostatic double layer at the water/lipid interface. Incorporation of cations and dications changes the properties of the lipid bilayer such as diffusion, viscosity, and the electrostatic pattern. At high ionic concentration, the bilayer acquires a long-wavelength standing undulation, corresponding to a change of phase from fluid planar to ripple. All these changes are potentially able to affect processes relevant in the context of cell biology. The major difference between cations and dications concerns the kinetics of absorption, which takes place nearly two times faster in the [P_6,6,6,6_]^+^ case, and for [DxC10]^2+^ dications displays a marked separation into two-stages, corresponding to the easy absorption of the first phosphonium head of the dication and the somewhat more activated absorption of the second phosphonium head of each dication.

## I Introduction

The constant threat of disease and the recurrent emergence of resistant bacterial strains^1,2^ motivates the search for ever new antimicrobial compounds. The quest is far from over, since a variety of therapeutic needs are still unmet, but the combined effort of the past few years led to the discovery of several new families of antimicrobial agents, consisting either of small molecules (for instance: steroids^3^), or oligomeric/polymeric species (peptides,^4,5^ among others). Most of these compounds share a cationic character, a fact often rationalized in terms of the negative surface charge of most bacteria at physiological conditions. A few year ago, the attention was briefly sparked by the discovery of a new family of closely related compounds, collectively known as phosphonium dications,^6^ consisting of two tetra-alkyl phosphonium cations [P_*ijkl*_]^+^ linked by an aliphatic chain (−C_*n*_−) made of *n* −CH_2_– monomers, displaying promisingly high antimicrobial and low cytotoxic activity. Several of these compounds, which can be synthesized on a large scale from commercially available precursors, are stable and liquid at room temperature,^6^ and thus belong to the vast class of room temperature ionic liquid (IL, for short) compounds.

**Figure 1:**
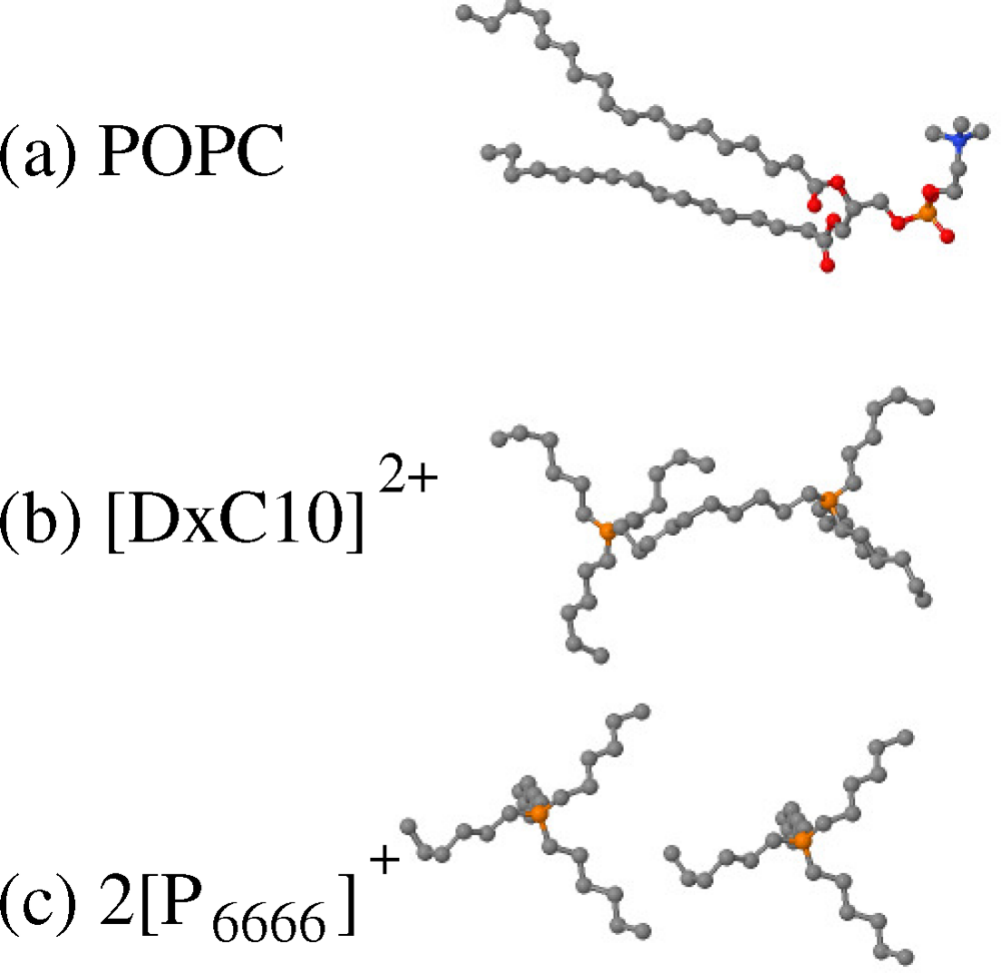
Structure of (a) the POPC molecule, (b) the [DxC10]^2+^ dication, and (c) the [P_6,6,6,6_]^+^ cation. All species are drawn to scale. Key: black dots: C; red dots, O; orange dots, P. Hydrogen atoms are not shown.

According to ref (6), phosphonium dication salts behave like broad spectrum antibacterial species, active at low micromolar concentration against both Gram-negative and Gram-positive bacteria. In the cases tested in ref (6), these compounds kill bacteria (i.e., not only inhibit them), and the bactericidal action is fast, taking between 15 to 30 min to kill drug-resistant strains of *Staphylococcus aureus*, as well as *Escherichia coli*. Among the tested species, 1,1′-(1,10-decanedyl)bis[1,1,1-trihexyl] chloride ([DxC10][Cl]_2_), whose [DxC10]^2+^ cation is made of two trihexyl phosphonium ions joined by a −(CH_2_)_10_– alkyl chain (see Figure 1), presented the strongest bactericidal activity, together with the weakest (or at least the slowest) cytotoxicity. These appealing properties stand out in comparison with those of similar compounds. For instance, monophosphonium species, as well as the corresponding ammonium species, are bactericidal as well as cytotoxic;^7,8^ therefore the real novelty of [DxC10][Cl]_2_ is that this bactericidal dication is not severely toxic to mammalian cells, opening the way to pharmaceutical applications. This specificity points to some peculiar mechanism of action for [DxC10]^2+^, and to a sizable margin for optimizing its antimicrobial properties by structure–activity studies covering broad families of related compounds. In interpreting these observations, however, one should also consider that selectivity in the biological effect might also arise from structural and water solvation properties (i.e., *nanostructuring*) of the ionic liquid itself,^9^ independent of any specific interaction of [DxC10]^2+^ with biomolecules.

Although remarkable, the biological relevance of phosphonium dications is not a complete surprise, since, for instance, [DxC10]^2+^ is a special case of the broad bolalipids (or bolaamphiphiles) family,^10,11^ whose effect on the stability and permeability of biomembranes has been extensively investigated.^12,13^ Bolalipids belonging to the phospholipid class, in particular, are a distinctive component of archaea; hence, they might have played a role in biosystems since the early stages of life on Earth.

The general context in which the observations of ref (6) find their natural setting could be summarized as follows. Most of the available experimental information on the effect of IL on cells is of macroscopic character, arising from bioassays and cell morphology studies (see, for instance, ref (14)), or from thermodynamic (calorimetry, water/octanol partitioning) measurements. In particular, the majority of studies consist of screening broad families of IL compounds for their effects on model lipid bilayers and/or cell lines, while the mechanisms underlying the observed effects are only sporadically discussed. Nevertheless, accumulated evidence shows that cationic antimicrobial agents kill bacteria or inhibit their growth by a variety of molecular mechanisms,^9,15,16^ which are the same ones that also determine their unwanted cytotoxicity. In some cases, the effect is due to the disruption of the lipid and/or protein components of the cell biomembrane,^17,18^ which changes its permeability with respect to metabolites. In other cases, the damage may occur deeper inside the cell. In eukaryotic cells, for instance, cytotoxicity might be due to the disruption of the biomembrane of subcellular organelles such as the nucleus, mitocondria, or chloroplasts, affecting crucial enzymatic and energy transformation processes. In all cell types, a potentially relevant toxicity mechanism might concern the alteration of replication processes, driven by the strong interaction of cations with highly anionic nucleic acids. The antibacterial activity of phosphonium cations, however, is generally attributed to their effect on the cell biomembrane.^19^ Moreover, the positive correlation between toxicity and lipophilicity in phosphonium and ammonium cations suggests (but does not strictly prove) that the first relevant interaction occurs between the cation and the lipid bilayer fraction of the biomembrane.

A general trend in the exploration of pharmaceutical properties of IL is their incorporation into polyelectrolytes. The advantages are primarily represented by increased stability and tunability, and by the potential to achieve higher local charge densities. Biological assays, for instance, show in some cases an increase of the effect (both bactericidal and cytotoxic) when measuring the dosage by the density of repeated units. Joining cations into dications seems to deviate from this trend, as several studies confirm that the lower cytotoxicity of dications with respect to the monovariety is a general property.^14,15,20^

Besides these macroscopic data, limited structural information at the microscopic or at intermediate length scales is provided by NMR, and by electrochemical ζ-potential measurements (see, for instance, ref (21)) and also by analysis of the luminescence of suitable chromophores. Neutron^22^ and X-ray^23,24^ reflectometry have been used to provide structural information on lipid-IL systems, achieving subnanometer resolution in the direction orthogonal to the bilayer, although averaging in the bilayer plane.

In principle, an atomistic view of IL interacting with biomembranes could be provided by computer simulation and by molecular dynamics in particular. Activity on this subject, however, has been limited, and focused primarily on the interaction between IL and lipid bilayers, which admittedly represent a very idealized model of biomembrane.^17,25−28^ Moreover, the number of available studies is minuscule compared to the variety of systems and conditions. Not surprisingly, only a handful of papers have investigated the difference between mono- and dicationic species.^29^ Moreover, most of the MD studies have been devoted to the popular imidazolium salts, while other IL families are much less covered. For instance, very limited information is available on IL based on phosphonium cation and lipid membranes from atomistic models simulated by molecular dynamics.^30^ The general aim of microscopic studies has been the relation of size, shape, and flexibility of organic ions with antibacterial properties and cytotoxicity toward eukaryotic cells.^31^

The present study aims at comparing the interaction of di- and monocations with palmitoyl–oleoyl–phosphatidylcholine (POPC) bilayers, representing a very idealized model of the biomembrane. Bacterial biomembranes have a clear anionic signature, while mammalian ones are primarily zwitterionic. We investigate the zwitterionic POPC precisely to gain insight into the (mild) effect of phosphonium dications on mammalian cells and also because the effect on POPC might represent a lower bound of the effect on more anionic lipid bilayers, similar to those present in bacteria. Last but not least, POPC is also an important component in the biomembrane of red blood cells (RBC), and hemolysis is often used as a proxy for cytotoxicity.^32^

Because of the vast difference between the real and simulated systems, our computations cannot address directly the most biological aspects of the organic mono- and dication salts interactions with cells or even biomembranes, but focus on chemical and biophysical aspects of the ternary water/lipid/IL, aiming at enlarging the limited knowledge available at the microscopic level on phospholipids and dicationic ILs, with potential impact in materials science, nano- and biotechnology, biomedicine, and drug delivery.

The results of room temperature MD simulations covering microsecond time scales show how the dication moiety of the [DxC10][Cl]_2_ salt approaches and then becomes absorbed into POPC bilayers. Absorption is a two stage process: in the first stage only one phosphonium head per dication is absorbed into POPC, while on longer time scales also the second cationic head reaches the lipid subsurface. Over the microsecond simulation time scale, the dications remain confined to the lipid leaflet in which they have been absorbed, without jumping to the complementary leaflet. At absorbate concentrations of the order of 1 dication per 10 nm^2^, the lipid bilayer acquires a standing undulation of wavelength equal to the side (20 nm) of the simulation box. Absorption into POPC of the corresponding [P_6,6,6,6,_]^+^ monocation occurs in a similar way but, of course, without the two stages character. Moreover, the kinetics is approximately twice as fast, and the acquired undulation is more marked and more stable, resembling the ripple phase of POPC which, in neat water/lipid systems, is stable several °C below the gel–liquid main transition of POPC.

## II Model and Method

Systems consisting of POPC, water and phosphonium chloride salts have been simulated using molecular dynamics (MD) at *NPT* conditions, based on an empirical force field model for the interaction of particles at nearly atomistic detail. The force field for POPC, in particular, is from ref (33)., with the inclusion of atomic charges taken from ref (34). The molecular structure of the lipid is slightly coarse-grained by resorting to a united atom representation of the hydrocarbon tails of POPC, i.e., replacing each CH_*n*_ (*n* = 1, 2, 3) group by a single particle. Water has been modeled through the extended simple point charge (SPC/E) model.^35^ The phosphonium ions have been described with a force field belonging to the Gromos^36^ family (version Gromos53a6) whose all-atom parametrization has been obtained through a web-based utility.^37^ The same Gromos force field has been used also for the Cl^–^ anion. The quality of the force field for the single [P_6,6,6,6_]^+^ and [DxC10]^2+^ ions has been verified by a few density-functional computations carried out using the CPMD computer package,^38^ with a PBE exchange-correlation approximation, atom-centered empirical dispersion energies,^39^ and open boundary conditions (see the CPMD manual). For both ions, conjugate gradient minimizations have been carried out using both CPMD and Gromacs, comparing the geometry, stretching and bending energies of the low energy isomers. The potential energy profile along selected dihedral angles has been computed as well, finding qualitative and semiquantitative agreement between the ab initio and the empirical approach. More importantly, the compatibility of the different pieces of the overall force field has been verified by a few density-functional computations carried out on neutral [P_6,6,6,6_][Cl] and [DxC10][Cl]_2_ species in close contact with a POPC phospholipid. In this case, the geometry of the complex was determined by conjugate gradient using Gromacs, and the resulting structure was given in input to CPMD. In all cases, we verified that the Gromacs geometry did not deform significantly during the CPMD energy minimization, carried out again by conjugate gradient. No comparison has been made at this stage for small aggregates including water. Admittedly, the validation is far from systematic and is also rather qualitative, but a comprehensive comparison of the two potential energy surfaces is beyond the scope of our computation. Needless to say, no empirical force field of practical complexity will ever quantitatively reproduce the ab initio data, and both will differ from the exact Born–Oppenheimer surface of the system.

All simulations have been carried out with the GROMACS package,^40^ integrating Langevin equations of motion to enforce the constant temperature condition, with coefficients set to give a relaxation time of any temperature imbalance of 2 ps. Constant pressure is enforced through the (3D) Parrinello–Rahman algorithm, again with a relaxation time of 2 ps. The simulation cell, of orthorhombic shape and volume exceeding 2.4 × 10^3^ nm^3^ encompassing more than 1.5 × 10^5^ atoms, is periodically repeated in space. Long range Coulomb forces have been computed by the PME algorithm.^41^ The contribution of long-range dispersion interactions to the energy and pressure of the system have been added using the isotropic correction provided by Gromacs. The isotropic estimate contrasts with the layering of the sample, but water and POPC have similar densities of valence electrons, and their average ⟨*C*_6_⟩ coefficient (see the Gromacs manual^40^ for the definition) is not very different. More importantly, the estimation of these contributions by a more detailed approach such as PME (also available in Gromacs) adds significantly to the cost of an already sizable computation.

For the sake of definiteness, in what follows, the *xy* plane will correspond to the approximate plane of the lipid bilayers; hence, *z* is normal to the bilayers. The analysis of MD trajectories relied on a variety of tools, from standard visualization tools available in VMD^42^ and in Jmol^43^ to simple homemade tools to compute diffusion constants, density profiles, electrostatic potentials, number and distribution of hydrogen bonds, etc. The diffusion constant of water, lipids, and ions absorbed into lipid, for instance, is computed using the Einstein formula in its 2D form:
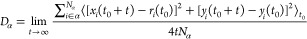
1where (*x*_*i*_, *y*_*i*_) represents the component of the position vector **r**_*i*_ in the *xy* plane, *N*_α_ is the number of molecules of species α, and ⟨···⟩_*t*0_ indicates averaging over *t*_0_. The 2D formulation is adopted despite the deviations from planarity of the average bilayer configuration, since for all species the motion along *z* remains bounded from below and from above, and thus it does not contribute to diffusion.

**Figure 2:**
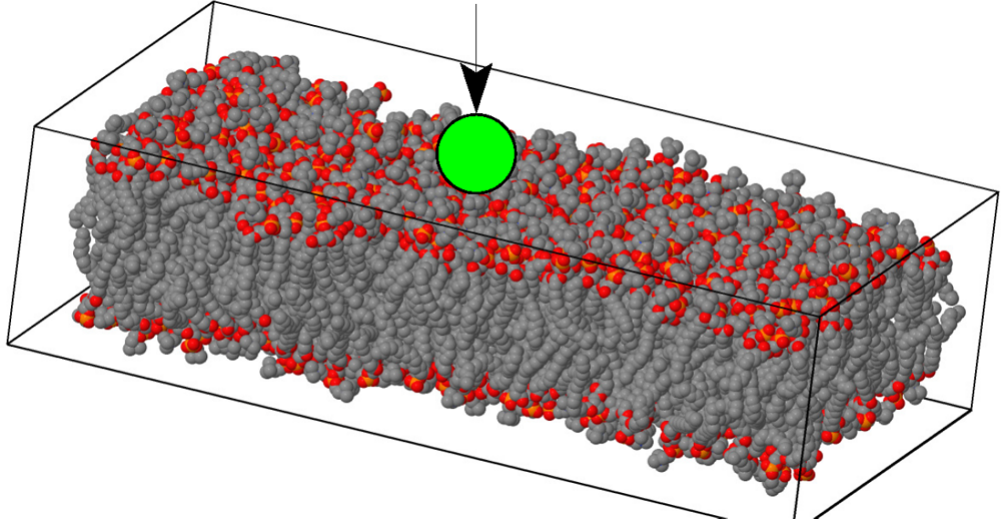
Schematic representation of the virtual-AFM introduced in Section II. A spherical probe of radius *r*_0_ (large green particle) is lowered toward the lipid bilayer until first contact with one of the lipid atoms. If, at contact, the center of the probe is at (*x*,*y*,*z*), the surface will have height *z* – *r*_0_ at in-plane coordinates (*x*,*y*). The probing sphere is not drawn to scale: in the computation, its radius *r*_0_ = 2 Å is comparable to the atomic radii. Water does not enter the surface determination, and it is not shown in the figure.

The equilibrium and time dependent layering of the various species along the average plane *xy* has been characterized by a further tool, that for convenience we name virtual AFM (vAFM). The idea is so intuitive that certainly it has been introduced and used many times in the literature, and, in the past, it has been used at least by our group for similar analyses. In this study, however, it plays a major role to provide a discretized representation of surfaces delimiting the distribution of lipids, water and ions. Let us consider for instance the definition of the two instantaneous surfaces {*z*_α_(*x*, *y*), α = *upper*, *lower*} limiting a POPC bilayer in any given configuration at time *t* along the MD trajectory. First, a radius is assigned to each POPC atom, according to a standard table of van der Waals radii.^44^ Then, to define, for instance, the upper surface (see Figure 2), a test particle of radius *r*_0_ = 2 Å is inserted well above the surface and lowered in short steps *δz* until the first distance separating the center of the test particle and a POPC atom becomes equal (in practice slightly less) to the sum of their two radii. If the contact occurs when the center of the test particle is at (*x*_*i*_,*y*_*i*_,*z̅*), the point identified on the surface is assumed (by definition) to have coordinates {*x*_*i*_,*y*_*i*_,*z*_*upper*_(*x*_*i*_,*y*_*i*_) = *z̅*–*r*_0_}. The definition of a point on the lower surface is analogous, with the obvious difference that the test particle is moved upward, and the location of the surface with respect to the contact point (*x*_*i*_,*y*_*i*_,*z̅*) is {*x*_*i*_,*y*_*i*_,*z*_*lower*_(*x*_*i*_,*y*_*i*_) = *z̅* + *r*_0_}. The reason for assigning a small but nonvanishing size to the test particle is to provide a first measure of local smoothing, while the *r*_0_ offset between the contact point and the corresponding surface point is to avoid a systematic overestimation of the (in this case) lipid volume due to the radius of the test particle. Exploiting the short-range of the test particle contact interaction, the determination of a point on the surface takes a small fraction of a second; therefore, it can be repeated many thousands or even hundreds of thousand times for each configuration of interest. The same procedure to identify the POPC/water interface, based on the coordinates of the POPC atoms only, is used also in the case of samples with dissolved phosphonium salts. In all cases, the instantaneous surface turns out to be rather rugged (see Figure S1 in the Supporting Information), showing an arrangement of bumps that, as we verified, correspond to individual lipids in the bilayers, thus confirming the high resolution of the scanning.

The point by point definition of surfaces is used in two major ways. On the one hand, the procedure is carried out on a regular 2D grid spanning the *xy* cross section of the simulation cell. This discretized representation of the surface can be interpolated and smoothed by Fourier analysis to provide a continuous geometric surface *z*_α_(*x*, *y*), as detailed below. On the other hand, the procedure can be used for the Monte Carlo estimation of the instantaneous volume of lipids in a given phase, computing and averaging the width of the corresponding bilayer over several thousands of random (*x*_*i*_, *y*_*i*_) points. For instance, the thickness θ(*x*_*i*_, *y*_*i*_) of the lipid layer at (*x*_*i*_, *y*_*i*_) can be computed as the difference
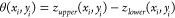
2Then, the average thickness of the lipid bilayer computed over *N* random points (*x*_*i*_, *y*_*i*_) is
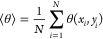
3The instantaneous volume is
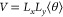
4where *L*_*x*_, *L*_*y*_ are the simulation box sides along *x* and *y*, respectively, for the configuration under investigation. The advantage of the random over a regular choice of points is that the error bar on ⟨θ⟩ and *V* can be determined by simple estimators for uncorrelated measurements. Moreover, and perhaps more importantly, the estimate can be improved by adding further random points without loosing the contribution of previous preliminary determinations.

To smoothen and interpolate the surfaces defined in the previous paragraphs, we resort to Fourier analysis. The samples used in the present simulation have an aspect ratio such that *L*_*x*_ ∼ 2*L*_*y*_. To obtain a Fourier representation of nearly equivalent resolution in the two directions of the (*xy*) plane, we use a number of real space points *N*_*x*_ along *x* which is double the number of points *N*_*y*_ along *y*. In particular, for all our samples, a grid of *N*_*x*_ = 256 and *N*_*y*_ = 128 has been used, corresponding to a grid spacing of about 1 Å in each of the two directions. The periodicity of the simulation cell discretizes the Fourier expansion to a sum of terms whose wave vector is of the form
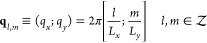
5Of these, only *N*_*x*_ × *N*_*y*_ (32768, in our case) terms can be independent, all the others being determined by the reciprocal space periodicity induced by the real space one.

Smoothing of the surfaces on the real space grid is obtained by expressing it as a reduced sum:
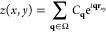
6Here *C*_**q**_’s are complex coefficients (with *C*_q_^*^ = *C*_–**q**_, since *z* is real), **r**_*xy*_ is the coordinate vector in the plane, and Ω is the set of **q** vectors whose norm is less than *q̅*, and whose number is somewhat reduced with respect to the original *N*_*x*_ × *N*_*y*_ ones. In the data reported below, the cut off is at *q̅*^2^ = 10 Å^–1^, corresponding to ∼16 000 complex waves, half of them independent, to represent the 32 768 real space points. In this way, the smoothed surfaces are close to the original ones, although the spikes that might be due to narrow holes or to single atoms or small groups sticking out of the surface are removed by the smoothing.

An important piece of information contained in the simulation trajectories is represented by the membrane-like fluctuations of the bilayer surfaces. These are measured by the time-dependent coefficients *C*_**q**_ of the Fourier coefficients in eq 6 of the wave-like excitations, whose mean square fluctuations at equilibrium carries information on thermodynamic quantities such as the water/lipid interfacial tension γ and bending rigidity *K*_*c*_, according to^45,46^
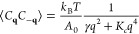
7where ⟨···⟩ indicates average over time or, equivalently, over the corresponding ensemble. Moreover, *A*_0_ is the projection of the POPC/water interface on the *xy* plane, equal to the simulation box cross section along the same plane. Since several of our samples are not planar even on average, we modified the expression into:
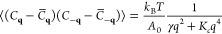
8where *C̅*_**q**_ = ⟨*C*_**q**_⟩. In this way, the left-hand side of eq 8 still measures the square modulus of fluctuations around the average surface position. The practical application of this relation, however, requires some additional care, as explained in the following section, Section III.

To be precise, the interfacial tension γ of a fluid bilayer at equilibrium in a fluid environment should vanish at equilibrium under no-stress conditions.^47^ The results of our simulation and previous simulations show that the γ of phospholipid/water interfaces is moderate but certainly not vanishing to within the estimated error bar, nor is it negligible, being about half of the surface tension of water at ambient conditions, The discrepancy with the predictions is due primarily to the fact that our samples represent stacks of hydrated bilayers, whose membrane-like fluctuations are correlated, constrained because of proximity with neighboring bilayers, and limited by the relative rigidity of the water interlayer. Moreover, the assumption of independent optimization of volume and area of the bilayer,^48^ which underlies the result of ref (47), is somewhat violated in our small periodic systems consisting of a correspondingly small number of molecular building blocks. Last but not least, the finite size of the simulation cell prevents the sampling of fluctuations of very long wavelength and possibly very low free energy cost. Nevertheless, it is apparent that the most relevant information is carried by the curvature force constant (bending rigidity) *K*_*c*_, whose determination by simulation through eq 8, however, is challenging and affected by a sizable error bar.

A further byproduct of the discrete Fourier representation is the computation of the surface area, accounting for the deformation from the planar surface. Assuming that *z*(*x*, *y*) is single-values (no hangouts, which are excluded by our constructive definition of the geometric surface), the area of the surface is^49^
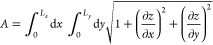
9This expression can be computed numerically on a regular grid, or, again on random points. As already stated, the surface of the lipid bilayers is rugged (see Figure S1 in the Supporting Information), therefore the area computed by eq 9 is significantly larger than its projection *A*_0_ on the (*xy*) plane.

A popular approximation, valid when corrugations represent only a small perturbation around the planar average, is
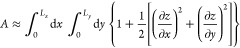
10hence:
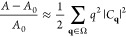
11In our systems, however, the results of this approximate relation contradict the assumption of low corrugation, and for this reason we will rely on eq 9 only.

The continuous definition of the surface allows us to count the number of atoms, ions, and molecules on each side of the surface of interest, as defined for the system and instantaneous configuration. The continuous surface allows also to make histograms of the density distribution with respect to the fluctuating surface, which is sometimes more telling than the distribution with respect to the average interface.

## III Results

**Figure 3:**
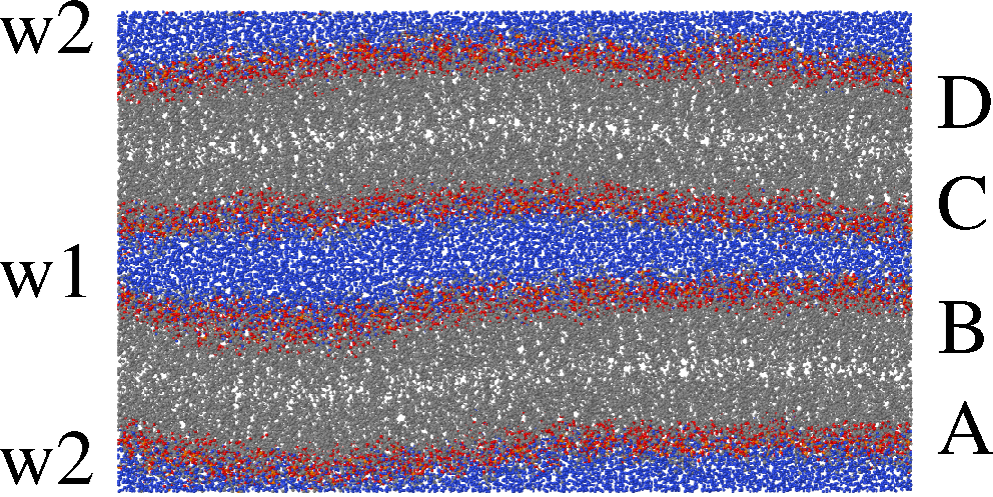
Side view of the simulation cell consisting of two parallel, rectangular lipid bilayers, separated by thin water interlayers. Key: black dots, C atoms in the POPC tail; red dots, O atoms in the polar POPC head; yellow dots, P atoms of POPC; blue dots, O atoms of water. Hydrogen is not shown. The capital letters on the right identify lipid leaflets whose properties upon cation absorption are discussed below. The two water interlayers are denoted as w1 and w2. The latter (w2) is split into two bands at the bottom and top of the figure by the application of periodic boundary conditions. In all but one case (sample VIII), the phosphonium salt is added in w1 only.

Computations have been carried out for samples representing stacks of planar phospho-lipid bilayers lying in the *xy* plane, hydrated by about ∼20 water molecules per lipid. More precisely, each sample contains 1360 POPC lipids arranged on two parallel, rectangular bilayers. The relatively low hydration level mimics the composition of phospholipid/water samples used in many neutron scattering experiments. The four distinct lipid leaflets will be denoted by the capital letters A to D, according to the scheme in Figure 3, which also labels the two water interlayers w1 and w2. The sample is enclosed into a periodically repeated orthorhombic cell whose thermally fluctuating periodicity *L*_*z*_ along *z* is such to accommodate two POPC bilayers (∼4 nm each) and two thin (∼2 nm each) water interlayers. The nearly 2:1 aspect ratio *L*_*x*_:*L*_*y*_ in the (*xy*) plane has been selected to provide a preferred direction (*x*) for the development of long wavelength modulations of the POPC bilayer, due to the effect of the electrolyte or to spontaneous breaking of planarity. The periodicity *L*_*x*_ along *x* is somewhat longer than but comparable to the wavelength of the ripple phase of POPC.^50^ The two water interlayers separating the POPC bilayers consist of ∼13500 water molecules each, the precise number depending on the sample. The hydrated POPC sample, illustrated in Figure 3 is stable in the planar liquid phase *L*_*d*_ (identified according to the criteria listed in ref (51)) over the time scale of the simulations described below, reaching up to 3 μs. Moreover, the same neat sample has been used in previous simulations by our group,^25,26^ and its accumulated simulation history far exceeds the duration of the present simulations. Over time, the two lipid bilayers in sample I fluctuate, due to thermal wave-like excitations, but they retain their planar geometry and their identity, since the (relatively incompressible) water interlayer effectively keeps them at nearly constant separation. In particular, over the entire simulation, no sizable variation of the lipid bilayer thickness occurs in the neat sample, and also no contamination by water of the inner hydrocarbon-tail range of the bilayer has been observed. In fact, in all snapshots of the neat sample, it is easy to distinguish the two water films from the layered stacking of the polar heads and of the electrostatically neutral hydrocarbon tails of POPC (see Figure 3).

Again for the neat POPC/water sample, the area *A* measured along the fluctuating interface, reported in Table 2 and computed according to eq 9, is much wider (+ 70%) than the area *A*_0_ of the bilayers projected on the (*xy*) plane. Both *A*_0_ and *A* increase slowly with increasing *T* (see the POPC/water data at *T* = 280, 300, and 320 K in Table 2), in line with the overall thermal expansion of the POPC/water sample, with *A* remaining ∼70% larger than *A*_0_.

The primary aim of simulations has been to investigate the effect of geminal, i.e., symmetric, dications on the structure and stability of POPC bilayers. In the dication simulation, the phosphonium species consisted of [DxC10]^2+^, whose structure is represented in Figure 1b, showing that its size and complexity is comparable to that of the POPC molecule (see Figure 1a). The water solvation properties and the formation of nanostructures in water by this same phosphonium dication have been discussed in ref (52). The concentrations considered here, however, are at the lower end of the range considered in ref (52). Moreover, the limited thickness of the water interlayers prevents a detailed investigation of [DxC10][Cl]_2_ nanostructuring in the water solution coexisting with the lipid phase. To highlight the peculiarities of the dication form, solutions of the monophosphonium [P_6,6,6,6_][Cl] salt have been considered as well. The structure of [P_6,6,6,6_]^+^ is illustrated in Figure 1c, where it has been drawn two times to show the near equivalence of [DxC10]^2+^ with two [P_6,6,6,6_]^+^. In all cases, the anion is represented by Cl^–^. Therefore, the ILs considered in this study consist of a (mildly) hydrophobic cation and hydrophilic anion. The list of simulated samples is given in Table 1. To simplify the exposition, in what follows we sometimes denote samples using the labels defined in Table 1. The numbers of ions reported in the Table 1 refer to the whole simulated sample, and they can be converted into relative wt % compositions and surface charge densities using the data in the same Table 1 and in Table 2.

**Table 1:** Correspondence between the Label Identifying the Samples, Their Composition, and Interfacial Properties^a^

label	sample	*T* [K]	phase	γ [mN/m]	10^20^ × *K*_*c*_ [J]	SimTime [μs]
I	POPC/water	280	P	49.5 ± 1	0.696 ± 0.13	0.988
		300	P	42.0 ± 0.8	0.438 ± 0.05	2.697
		320	P	35.5 ± 1.3	0.417 ± 0.03	0.992
II	POPC/water +2 [DxC10][Cl]_2_	300	P	42.6 ± 1.0	0.415 ± 0.09	1.397
III	POPC/water +30 [DxC10][Cl]_2_	300	P/R	50.3 ± 0.5	0.360 ± 0.03	0.892
IV	POPC/water +60 [DxC10][Cl]_2_	280	P	90.8 ± 3.5	0.445 ± 0.06	2.092
		300	R	65.9 ± 2.5	0.208 ± 0.04	3.488
		320	R	47.6 ± 3	0.113 ± 0.05	1.996
V	POPC/water +4 [P_6,6,6,6_][Cl]	300	P	41.8 ± 0.9	0.420 ± 0.08	1.256
VI	POPC/water +12 [P_6,6,6,6_][Cl]	300	P	44.8 ± 0.8	0.281 ± 0.03	0.888
VII	POPC/water +120 [P_6,6,6,6_][Cl]	300	R	56.3 ± 2	0.261 ± 0.03	2.781
VIII	POPC/water +240 [P_6,6,6,6_][Cl]	300	R	59.0 ± 2.5	0.340 ± 0.03	1.912

a γ is the bilayer/solution interfacial tension; *K*_*c*_ is the lipid bilayer bending rigidity. All samples contain 1360 POPC molecules hydrated by about 20 waters per POPC molecule. Phases are labelled ”P” for fluid planar, and ”R” for ripple. ”P/R” means that the phase identification is uncertain. The simulation time excluding a short local equilibration is reported in the column marked SimTime.

**Table 2:** Average Area ⟨*A*⟩ of the Water-Lipid Interface Computed by eq 9 for All the Simulated Samples^a^

system	*T* [K]	⟨*A*⟩ [nm^2^]	[nm^2^]	⟨*A*_0_⟩ [nm^2^]	[⟨*A*⟩ – *A*_0_]/*A*_0_
POPC/water	280	345.49	4.05	201.70	0.712
	300	347.13	4.10	202.95	0.710
	320	351.94	4.19	204.80	0.719
POPC/water +30 [DxC10][Cl]_2_	300	363.64	4.06	203.40	0.788
POPC/water +60 [DxC10][Cl]_2_	280	360.46	3.48	201.00	0.793
	300	381.31	4.68	202.90	0.879
	320	394.25	5.18	204.80	0.925
POPC/water +12 [P_6,6,6,6_][Cl]	300	351.08	4.52	203.70	0.724
POPC/water +120 [P_6,6,6,6_][Cl]	300	390.64	4.61	204.10	0.914
POPC/water +240 [P_6,6,6,6_][Cl]	300	399.94	6.75	203.30	0.968

a The standard deviation  is also reported. ⟨*A*_0_⟩ is the average interface area projected on the plane (*xy*), computed from the time fluctuating periodicities as ⟨*A*_0_⟩ = ⟨*L*_*x*_ × *L*_*y*_⟩. The error bar on ⟨*A*_0_⟩ is around 50 Å ^2^ for all samples. [⟨*A*⟩ – *A*_0_]/*A*_0_ represents the relative excess of *A* over *A*_0_ due to the interface roughness and long wavelength rippling.

Samples with phosphonium mono- and dications have been prepared starting from the well equilibrated water–POPC samples described in the previous paragraphs, adding closely spaced cation and neutralizing anion(s) in the central nm of the ∼2 nm wide water interlayer, at the same time removing closely overlapping water molecules. In all but one case (sample VIII), ions have been added into one (w1) of the two water interlayers only, making the two lipid leaflets in each bilayer inequivalent. Since the four leaflets in these samples are simulated together at the same (*P*, *T*) conditions, the difference between leaflets A and B, or between C and D, will highlight the specific effect of adding the salt. Moreover, the choice of adding ions into w1 only makes it easy to spot any jump by the absorbed cations across leaflets. Last but not least, the one sided penetration of cations into the lipid bilayers provides a better representation of the effect of ILs on cells, in which the added ions diffuse from the outside toward the cell inside, making the two leaflets inequivalent. The number of [DxC10]^2+^ dications in the simulated samples spanned the range from 2 to 60. The highest number corresponds to one dication every 11 POPC molecules in the leaflets B and C that surrounds the w1 interlayer, giving origin to a sizable surface charge density on these two leaflets. The number of [P_6,6,6,6_]^+^ ions in our simulations ranges from 4 to 240. In the 240-[P_6,6,6,6_][Cl] system (sample VIII), however, an equal number of ion pairs are inserted in the two water interlayers, resulting in the same surface charge density on each leaflet as in the two center-most leaflets of the [DxC10][Cl]_2_ sample of highest salt concentration. In the preparation of the POPC/salt/water samples, ions have been added in stages, with only a few (up to ∼10) neutral cation–anion units being added at each step. After each addition, the energy is minimized, and the systems briefly relaxed (∼100 ps) at *T* = 300 K, before adding a new set of ions until reaching the target mono- or diphosphonium concentration.

The progressive and slow addition of ions in the water interlayer(s) limited somewhat the instantaneous strength of the perturbation on the lipid bilayers, that remained virtually unchanged and free of ions for a short time while cations migrated toward the lipids, leaving anions well hydrated in the interlayer(s). We adopted this strategy of staged additions after observing the drastic deformation and poration through the bilayers due to the sudden addition of many charges, as shown in Figure S2 of the Supporting Information. Most of our simulations have been carried out at *T* = 300 K, but shorter simulations covering about 2 μs have been performed also at *T* = 280 K and *T* = 320 K. The main transition temperature of POPC is *T*_*m*_ = 270.5 K, both in experiments^53^ and in computations^54^ similar to the present ones. Less well documented is the pretransition giving origin to the ripple phase,^55^ which plays a role in the results of the present simulations. The wavelength in hydrated POPC samples was experimentally determined to be λ = 169 Å at *T* = 288 K,^50^ possibly in a slightly metastable state of the system.

### III.A The Low Salt Concentration Limit

**Figure 4:**
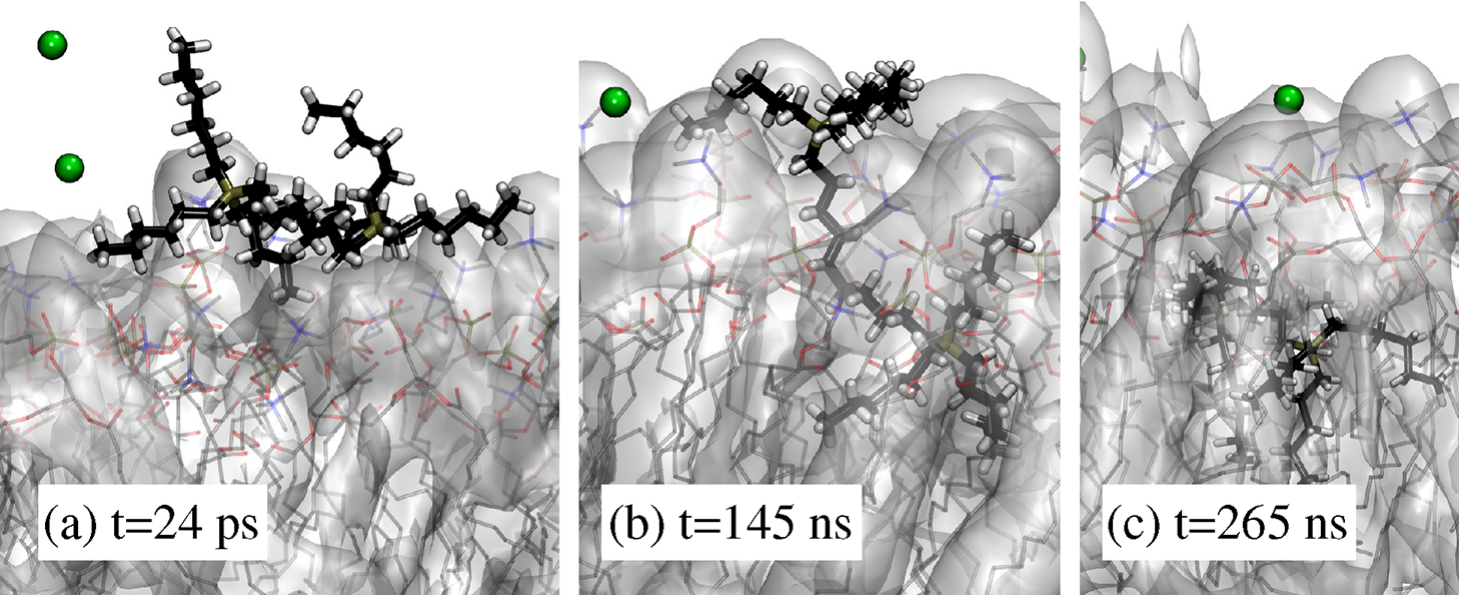
Sequence of snapshots from the simulation of virtually independent [DxC10]^2+^ and [Cl]^−^ ions in solution (system II in Table 1), illustrating the successive stages of one [DxC10]^2+^ entering the lipid layer. (a) The dication is adsorbed at the water/lipid interface, (b) one of the cationic heads entered the lipid phase, and (c) the whole dication is absorbed in the lipid phase. The dication structure is identified by the thick bond lines. Time is measured from the unrelaxed insertion of the ions in the w1 water interlayer.

Visualization of trajectories for samples with only two [DxC10]^2+^ or four [P_6,6,6,6_]^+^ neutralized by four [Cl]^−^ anions allows to define different stages in the absorption process of virtually isolated cations into POPC. Let us consider, for instance, the case of two [DxC10][Cl]_2_ molecules, added as dissociated ions. Despite its sizable mass, [DxC10]^2+^ has a non-negligible diffusivity in water (estimated in ref (52) at *D* = 6 × 10^–7^ cm^2^/s at 26 wt % salt in solution), possibly due to its predominantly hydrophobic character. Because of this high diffusivity, and of the limited width of the water interlayer, the two dications get quickly (on the order of several picoseconds) into contact with the exposed lipid surface, driven by the inhomogeneity and anisotropy of water at the water/POPC interface. The further progression of the dication toward the subsurface layer of the polar POPC head, however, is apparently not a downhill process, as instead it is in the case of the smaller and simpler butylmethylimidazolium [bmim]^+^ cation (see refs (25 and 26)). Instead, [DxC10]^2+^ remains adsorbed just outside the POPC surface during times of the order of 100 ns. Such a relatively long latency time points to a free energy barrier slowing down the cation penetration into the bilayer, probably associated with the difficult crossing of the lipid polar head range by the neutral alkane tails surrounding the phosphorus ion. Eventually, absorption under the water–lipid interface occurs in two inequivalent steps separated again by ∼100 ns, corresponding to the penetration of the first and second cationic head of [DxC10]^2+^ (see Figure 4). This qualitative description of the absorption process has been made quantitative by computing and plotting as a function of time the distance of all ions from the surfaces of the lipid bilayers, determined by the method detailed in Section II, as well as the number of ions absorbed below the same lipid surface, shown in Figures S3 and S4 of the Supporting Information.

**Figure 5:**
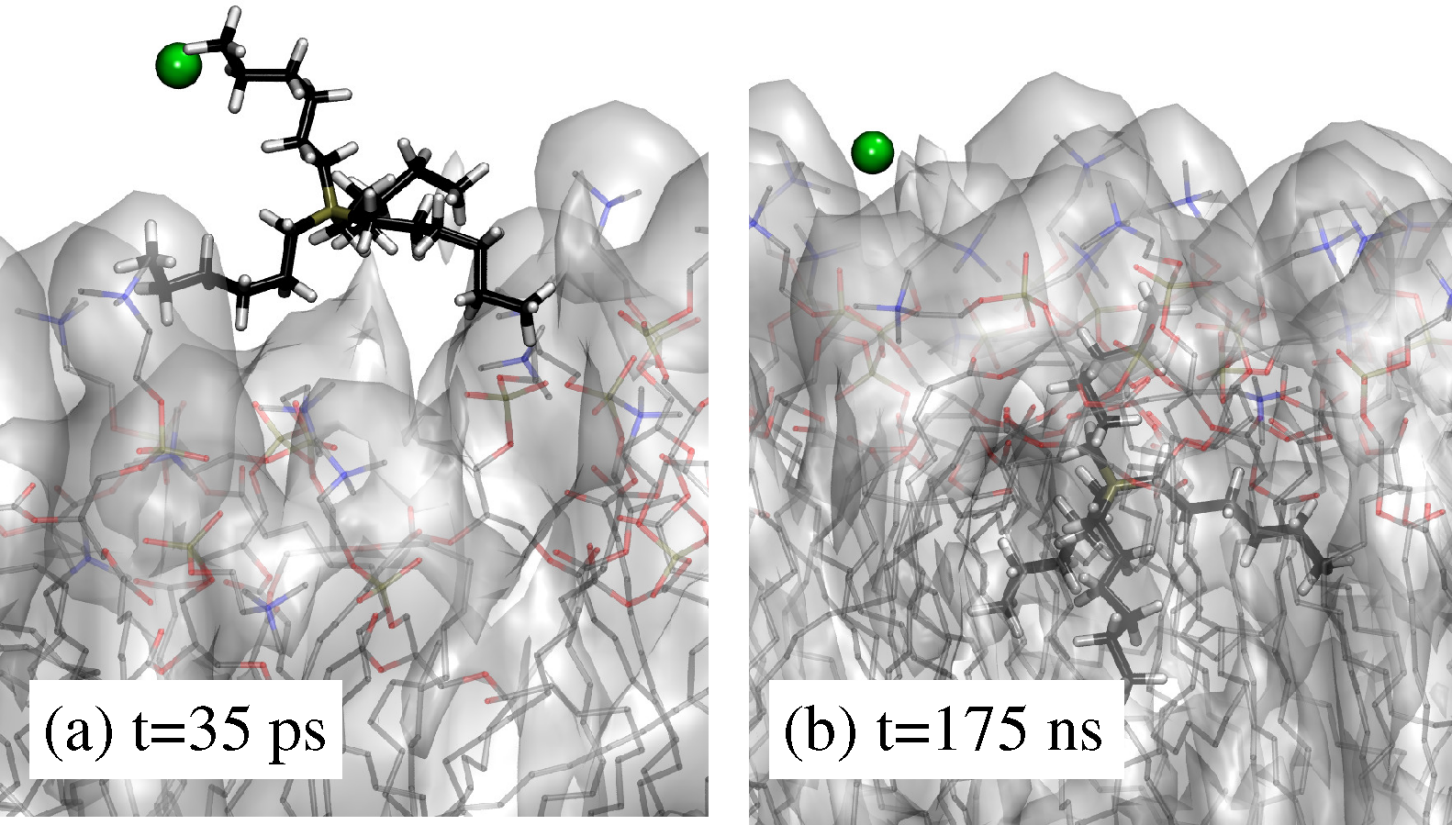
Two snapshots from the simulation of virtually independent [P_6,6,6,6_]^+^ and [Cl]^−^ ions in solution (system V in Table 1), illustrating (a) the [P_6,6,6,6_]^+^ cation just adsorbed at the water/lipid interface and (b) the same cation absorbed below the interface in the lipid phase. The [P_6,6,6,6_]^+^ cation is identified by the thick bond lines. Time is measured from the unrelaxed insertion of the ions in the w1 water interlayer.

The same type of visual and quantitative analysis shows that the time evolution of [P_6,6,6,6_]^+^ following its insertion into the water interlayer is similar to that of [DxC10]^2+^ (see Figure 5), but takes place on a faster time scale, and without the characteristic two-step kinetics of the dication case. Since the time to approach the lipid bilayer is already short, the faster diffusion of [P_6,6,6,6_]^+^ in water gives only a minor contribution to the enhancement of absorption. More relevant is the kinetics of [P_6,6,6,6_]^+^ when adsorbed onto the POPC surface, while it crosses through the zwitterionic skin of the POPC bilayer. On the time scale of simulation, the bilayers remain planar upon absorbing just a few di- or monocations. It might be useful to emphasize that the observations on the absorption kinetics are based on a single trajectory for each of the two IL samples. Despite the relatively small size of the ions, the cost of simulating the large bilayers and water interlayers prevents many repetitions of the basic insertion experiment, therefore the times measured on the single simulation trajectories remain as estimate of time scales.

It is important to remark that virtually no penetration into the lipid range by Cl^–^ has been observed in these low salt-concentration samples over several μs, suggesting that in higher concentration samples, the progressive absorption of many cations into the bilayer will result into the formation of charged interfaces, whose electrostatic energy might hamper the continuation of the process beyond the first few steps. We anticipate that the lipid/water interface in systems of high [DxC10]^2+^ or [P_6,6,6,6_]^+^ concentration will display marked rippling, that provides a way to increase the interfacial charge density while limiting the growth of the electrostatic energy, as it will be shown in the next subsection. This same effect, i.e., rippling, was already observed in ref (17), but attributed primarily to hydrophobic interaction and to the asymmetric distribution of cations between leaflets. Our simulations at medium-high salt concentration show that both electrostatic and hydrophobic interactions are likely to contribute to rippling and that, in our case, the asymmetric distribution of cations between leaflets is not required for rippling.

### III.B The Medium-High Salt Concentration Range

**Figure 6:**
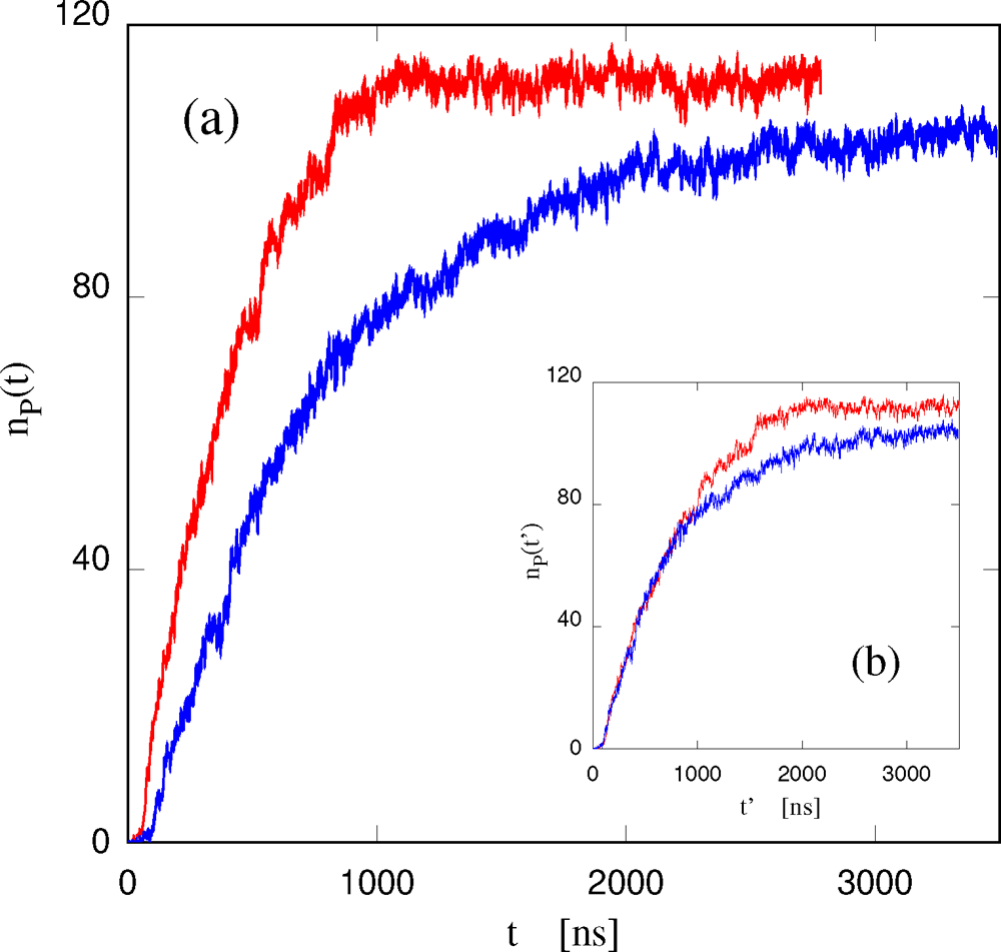
Number *n_p_* of [P_6,6,6,6_]^+^ cations (red curve) and cationic headgroups from [DxC10]^2+^ dications (blue line) absorbed into the lipid phase as a function of time. The inset shows the same curves plotted against the scaled time *t*′, where *t*′ = *t* for the dication and *t*′ = 1.88*t* for the monocation.

**Figure 7:**
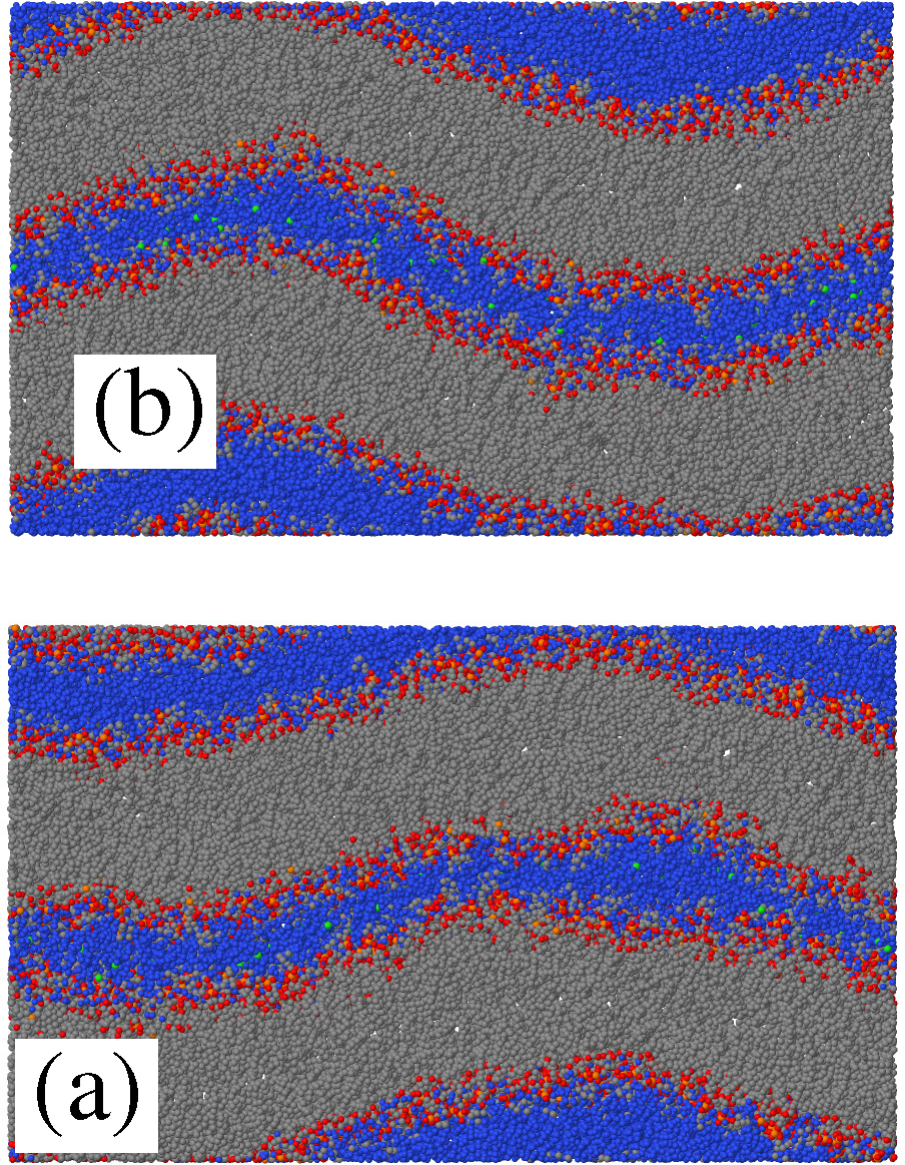
(a) Cross-section in the (*xz*) plane of the POPC+60 [DxC10][Cl]_2_/water sample IV at *T* = 300 K in the ripple phase. (b) Same for the POPC+120 [P_6,6,6,6_]][Cl]/water sample VII. Snapshots were taken after 3.5 μs of equilibration.

The number *n_p_* of cationic units absorbed into the lipid bilayers becomes statistically more significant with increasing number of ions in the sample, which provides a measure of self-averaging to all computed quantities. The time dependence of *n_p_* for the sample with 60 [DxC10][Cl]_2_, initially inserted into the w1 water intralayers, is reported in Figure 6a. The plot shows that (i) the absorption into the lipid bilayer progresses up to ∼90% of the cations initially placed in w1. The asymptotic value is estimated as the *t* → *∞* limit of the analytic fit described below. (ii) The saturation of *n_p_* to its equilibrium value requires several microseconds, a time which is long on the simulation time scale. (iii) A single exponential relaxation to the asymptotic value *n*_*p*_(*t* → *∞*) is unable to fit *n*_*p*_(*t*) over the entire time range. A quantitative fit requires two relaxing exponentials, according to the analytical expression:
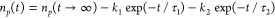
12where *n*_*p*_(*t* → *∞*), *k*_1_, *k*_2_, τ_1_, and τ_2_ are adjustable variables in the fit of the simulation data. The optimal time constants turn out to be τ_1_ = 0.54 μs and τ_2_ = 1.33 μs, with the crossover between the fast and the slow stages taking place at τ ∼ 1 μs. These results, in turn, show that [DxC10]^2+^ absorption does not follow a single step kinetics; analysis and animations of trajectories shows that the fast process corresponds to the absorption of the first cationic head of each dications. Only after completion of this first step, i.e., beyond *n*_*p*_ ∼ 60, the second absorption stage fully develops, corresponding to the absorption of the second cation head of [DxC10]^2+^. The switch over corresponds also to a change of the system morphology, in which the bilayers develop a long wavelength undulation (see Figure 7). (iv) [Cl]^−^ anions remain in water solution, causing a sizable charge separation in the sample, which eventually drives the rippling of the bilayers.

The results for the corresponding sample with 120 [P_6,6,6,6_][Cl] ion pairs are also shown in Figure 6a. It is apparent that the time evolution is qualitatively similar, but also, in this high salt-density case, the absorption kinetics of [P_6,6,6,6_]^+^ is faster than that of [DxC10]^2+^. More importantly, in the monocation case, a single exponential relaxation
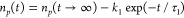
13of time constant τ_1_ = 0.32 μs is sufficient to fit the approach of *n*_*p*_(*t*) to its asymptotic value, which, moreover, is slightly higher (94% of inserted [P_6,6,6,6_]^+^, instead of the 90% of cationic heads for [DxC10]^2+^, estimated again from the analytic fit). Once again, [Cl]^−^ remains in water solution, as shown by the results of Figure S5 of the Supporting Information.

As already stated, most computations have been performed at *T* = 300 K, but more limited simulations for [DxC10][Cl]_2_ have been carried out also at *T* = 280 K and *T* = 320 K, from which preliminary insight on the temperature dependence of the absorption can be gained. As expected, relaxation times are longer at *T* = 280 K than at *T* = 300 K, and we have been unable to approach the late stages of [DxC10]^2+^ absorption into POPC, whose unbiased exploration would require simulation times in excess of 10 μs. However, a fit of *n*_*p*_(*t*) data over a time interval of ∼2 μs, using the same approach described above, allows us to estimate the asymptotic value lim_*t*→*∞*_*n_p_* = 70% of the added cationic units (i.e., each of the phosphonium heads in [DxC10]^2+^, well below the 90% observed at *T* = 300 K. Over the 2 μs simulations, moreover, the bilayer is planar and fluid, and most of the dications that are closely associated with the bilayer have only one cationic head absorbed below the lipid/water interface. In a qualitative and preliminary way, it is tempting to conclude that 50–60% absorption, with (mostly) one cationic head absorbed per dication is the limiting absorption capability of planar POPC bilayers at *T* = 280 K. The results for *T* = 320 K are simpler to interpret, and the 2 μs simulation almost reaches the asymptotic state for the 60 [DxC10][Cl]_2_ concentration we used. The fit of *n*_*p*_(*t*) at *T* = 320 K shows that the slow and the fast absorption processes are both ∼20% faster that at *T* = 300 K, and the asymptotic value of *n*_*p*_(*t*) reaches 94%, slightly higher than at *T* = 300 K and, probably by coincidence, equal to the value for [P_6,6,6,6_]^+^ at *T* = 300 K. The temperature dependence of the results shows that, as expected, entropy is a not-negligible driving force for the absorption of [DxC10]^2+^ into POPC. Since our simulations concern primarily the partitioning of [DxC10]^2+^ between water and POPC at equal total composition, every statement on entropy is necessarily a relative conclusion. In particular, from our data on the temperature dependence, we can say that, with increasing *T*, [DxC10]^2+^ gains more (or looses less) entropy when absorbed into POPC instead of being solvated in water.

Further insight on the relation of the absorption kinetics of the two homologous phosphonium species could be gained by relying on the scaling laws connecting their diffusion properties, irrespective of the medium (water, lipids, or pure ionic liquid phase) in which diffusion occurs. Then, a semiquantitative analysis could go as follow. The dication has a mass that is nearly twice that of [P_6,6,6,6_]^+^, and a radius that scales as *M*^1/3^ (compact aggregate). To lowest order, the prediction of the Chapman–Enskog theory is  (see ref (56)), while Stokes relation predicts the dependence on radius tobe *D* ∼ *R*^–1^. On the basis of these lowest order estimates, the diffusion coefficient of [P_6,6,6,6_]^+^ is expected to be (2^1/2^ × 2^1/3^ =) 1.88 times that of [DxC10]^2+^. The estimate is only semiquantitative, and does not account for the specific changes of diffusion coefficient following the cations absorption into the bilayer (see below), but the result is still useful, and it shows that diffusion kinetics might be the single most important factor in determining the time evolution of absorption, at least up to some high density of absorbed cations. In fact, it turns out that it is possible to superimpose the [DxC10][Cl]_2_ and the [P_6,6,6,6_][Cl] absorption curves by such a time scaling over a wide time interval of ∼1 μs (see Figure 6b). In other words, the number of absorbed [P_6,6,6,6_]^+^ ions at time *t* is equal to the number of P atoms from [DxC10]^2+^ absorbed under the surface at time 1.88*t*. Beyond 1 μs, the two curves deviate from each other. The reason for the slower rate is that the absorption of the second P-headgroup faces a higher barrier than the first one, and the difference might depend on the length of the linker. Nevertheless, eventually both [DxC10]^2+^ and [P_6,6,6,6_]^+^ reach virtually the same level of subsurface absorption, with only a slight (∼4%) advantage for [P_6,6,6,6_]^+^. The kinetics of absorption in sample VIII with 240 [P_6,6,6,6_][Cl] ion pairs absorbed into four leaflets is remarkably similar to the one of sample VII with 120 [P_6,6,6,6_][Cl] ion pairs absorbed into two leaflets.

Over the duration of simulation, no monocation or even phosphonium headgroup in [DxC10]^2+^ is seen jumping from the absorption leaflet to the complementary one in the same bilayer, which is in contact with the neat w2 water interlayer. On the one hand, jumps of this kind are bound to occur over longer times. On the other hand, jumps are not likely to equalize the density of cations in the two leaflets, because [Cl]^−^ anions are likely to remain in the w1 water interlayer even for much longer times, up to macroscopic scales.

**Figure 8:**
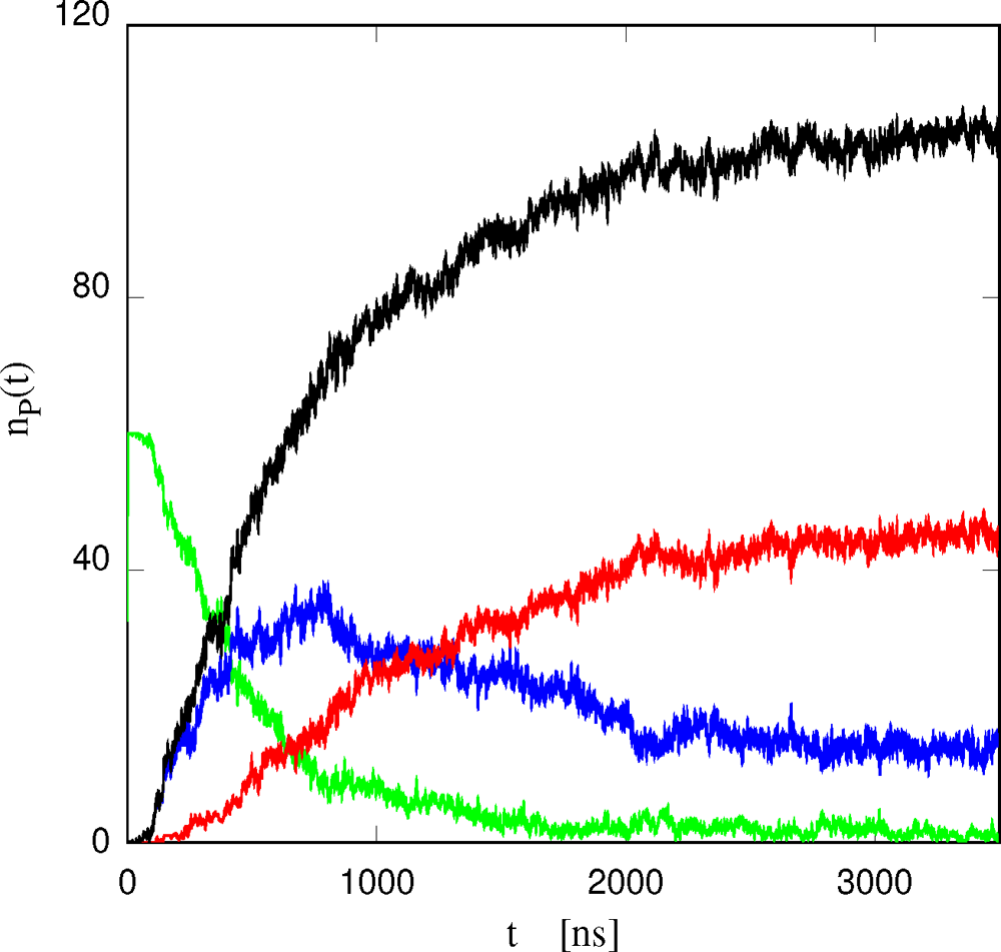
Green curve: number *n*_0_ of [DxC10]^2+^ ions contributing no P ion to the total number of absorbed P head groups shown in Figure 6; Blue curve: number *n*_1_ of [DxC10]^2+^ ions contributing one P to the same absorbed total. Red curve: number *n*_2_ of [DxC10]^2+^ ions contributing two P to the same absorbed total. Black curve: total number of absorbed P ionic centers, same as shown in Figure 6, given by *n*_1_ + 2*n*_2_.

The relevance of the dication structure in determining the two-stage absorption kinetics of [DxC10]^2+^ is emphasized by the analysis of Figure 8, showing the number of dications (out of 60) having no (*n*_0_), one (*n*_1_), and two (*n*_2_) phosphonium head groups absorbed in the lipid bilayers. As apparent from the figure, the absorption of cations starts ∼100 ns after their insertion into w1. At first absorption is virtually limited to one headgroup at most for cation, and only somewhat later the second headgroup of half absorbed cations enters the lipid phase. At time ∼1 μs, the number of cations having only one absorbed headgroup levels off, and starts to decrease, since the second headgroup is also being absorbed. At this same time, *n*_1_ = 2 × *n*_2_, and the overall absorption kinetics starts to slow down, suggesting a relation between these nearly simultaneous events.

**Figure 9:**
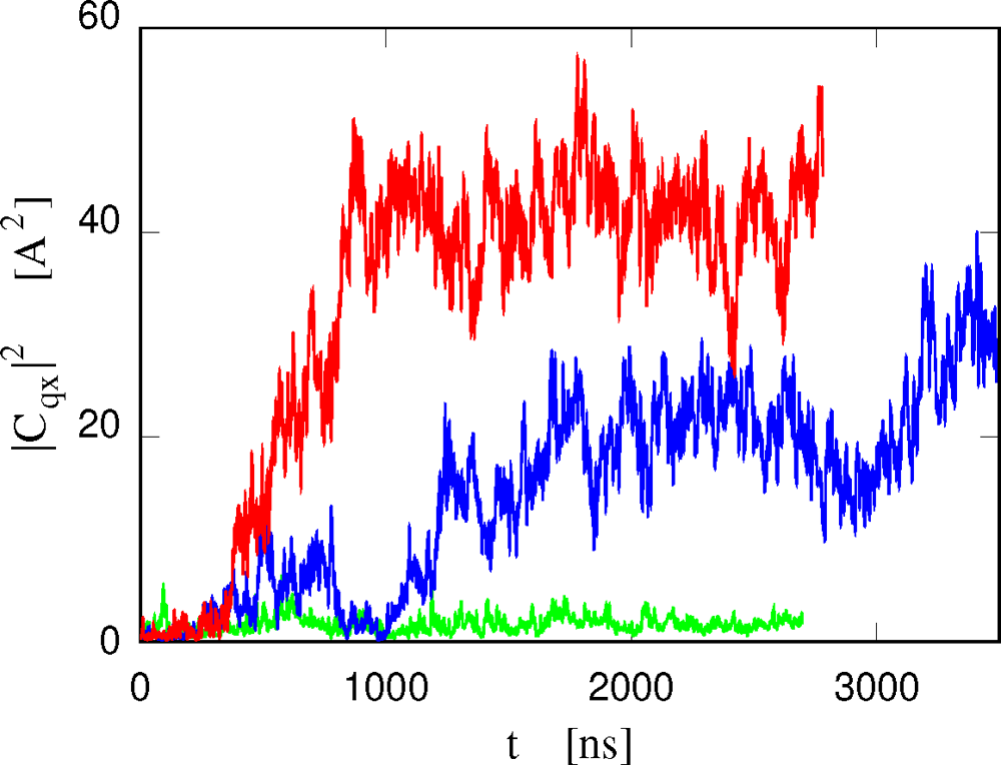
Square amplitude of the corrugation whose wave vector is **q**_*x*_ = 2π**x̂**/*L*_*x*_, where **x̂** is the unit vector and *L*_*x*_ is the periodicity along the *x* direction. The corresponding wavelength *L*_*x*_ is equal to about 12 nm. Red line: neat POPC bilayers in water. Blue line: sample IV (with 60 [DxC10][Cl]_2_). Green line: sample VII (with 120 [P_6,6,6,6_][Cl]). A mild smoothing has been applied to help visualize the trends.

To be precise, a further absorption milestone occurs at nearly the same time, corresponding to the rippling of the lipid layers in the POPC+60 [DxC10][Cl]_2_ sample, as will be apparent from Figure 9 discussed below. From the simulation alone, it is impossible to unambiguously distinguish cause from effect among these related events. At the end of the simulations, both the sample with 60 dications and the one with 120 phosphonium cations are stable in the ripple phase, as shown in Figure 7. The same figures shows that, because of the obviously strong coupling of leaflets in the same bilayer, rippling has virtually the same amplitude in all leaflets.

The progressive formation of a standing ripple in the bilayers with increasing absorption of cations in the lipid phase is documented in Figure 9, displaying the time dependence of the square amplitude |*C*_*qx*_|^2^ of the longest wavelength oscillation compatible with the periodicity of the simulation cell, having wave vector **q**_*x*_ = 2π**x̂**/*L*_*x*_, in three different samples. The neat POPC/water sample is simulated at stationary conditions; hence |*C*_*qx*_|^2^ displays fluctuations whose amplitude does not vary systematically with time. The amplitude |*C*_*qx*_| is small (on the order of an Å), consistent with the fluid planar phase of the sample. More interesting are the results for the phosphonium-added samples. In the POPC + 120 [P_6,6,6,6_][Cl]/water sample, for instance, the amplitude of the longest wavelength corrugation starts to increase after about 350 ns, a time at which nearly half of the added cations have been absorbed into the lipid bilayers. The full development of the ripple takes another 500 ns, after which the amplitude of the leading wave in the sample remains stationary. The ripple development is far slower in the POPC+60 [DxC10][Cl]_2_/water, consistently with all other observations on the kinetics of the two high salt-concentration systems. Despite some wide-amplitude fluctuations, the formation of an irreversible ripple starts at ∼1 μs, a time at which, as already stated, most dications are half adsorbed into the lipid bilayers, and the absorption kinetics turns into its slowest mode.

The area *A*_0_ of the bilayers projected on the *xy* plane, is not significantly affected by the addition of ions (see Table 2) or by the related transition to a ripple phase. The area *A* measured along the instantaneous corrugated lipid/solution interface, instead, shows a marked dependence on the sample composition and planar versus ripple structure. The major effect on *A* due to the salt addition is in fact due to rippling, which increases the excess of *A* with respect to *A*_0_ from the 70% of the neat POPC/water system to the 90% of the higher salt concentration samples. In the case of the salt-added samples, the effect of temperature is significant, but still mediated by the rippling transition. For instance, in the 60-[DxC10][Cl]_2_ sample, the bilayers transform from planar to ripple in going from *T* = 280 K to *T* = 300 K, and the excess in *A* over *A*_0_ increases from 79% to 88%. The equivalent change of temperature from *T* = 300 K to *T* = 320 K, over which the bilayers remain rippled, brings about a more limited change of [⟨*A*⟩ – ⟨*A*_0_⟩]/*A*_0_, increasing from 88% to 92%. At equal surface density of cationic charge, the effect of [P_6,6,6,6_][Cl] on rippling and increase of ⟨*A*⟩ is qualitatively and quantitatively similar to that of [DxC10][Cl]_2_. Absorbing cations on one or both leaflets has nearly the same effect, as shown by the comparison of the average interfacial area of lipids in sample VII and sample VIII.

**Figure 10:**
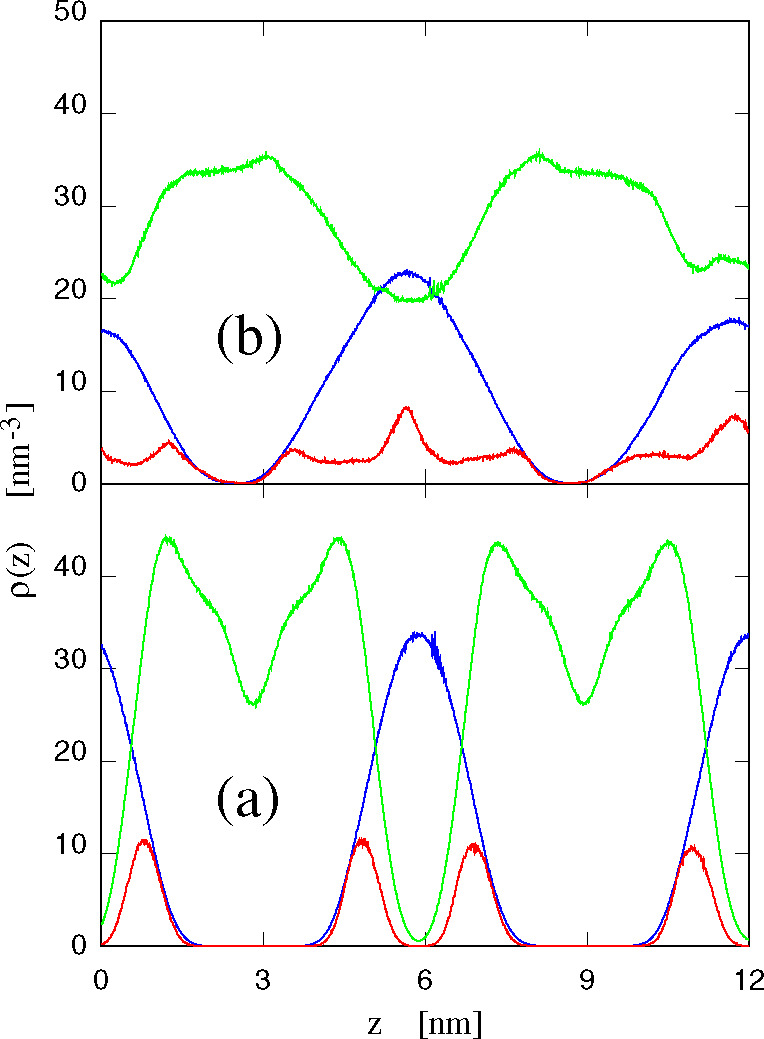
Number density distribution of non-hydrogen atoms along the direction *z* orthogonal to the average lipid/water interface. Blue line: water; green line: POPC; red line: P of POPC (which is counted also in the green line). The number density distribution of P atoms has been multiplied by 5 for clarity. Panel a refers to POPC in water (system I) at *T* = 300 K; panel b refers to POPC+60 [DxC10][Cl]_2_ in water (system IV) at *T* = 300 K.

Once the samples reach their equilibrium state, a simple characterization is provided by the density profile of the different species, i.e., lipids, water, and ions, along the direction *z* orthogonal to the average plane of the bilayers. The result, however, provides an immediate picture of the structural organization of bilayers only in the case they are in the fluid planar phase. In the ripple phase, instead, the profiles are much less easy to interpret. An example is provided in Figure 10, showing the number density profile of non-hydrogen atoms belonging to POPC and to water, computed along the *z* direction for (i) system I, i.e., POPC in water, in the planar fluid phase at *T* = 300 K, and for (ii) system IV (POPC+60 [DxC10][Cl]_2_) also at *T* = 300 K in the ripple phase. The figure shows that the relatively narrow water distribution corresponding to the two planar interlayers of the neat sample (Figure 10a) becomes much broader following the addition of [DxC10][Cl]_2_ (Figure 10b). Similar changes affect the distribution of atoms belonging to POPC, which in the neat case presents sharp interfaces and a minimum at the center of each bilayer, due to the defective packing of the disordered tips of the POPC tails. The largest qualitative change might be seen in the distribution of P atoms belonging to POPC, since the minima at the center of two interlayers turn into maxima in the rippled sample with [DxC10][Cl]_2_ in it.

**Figure 11:**
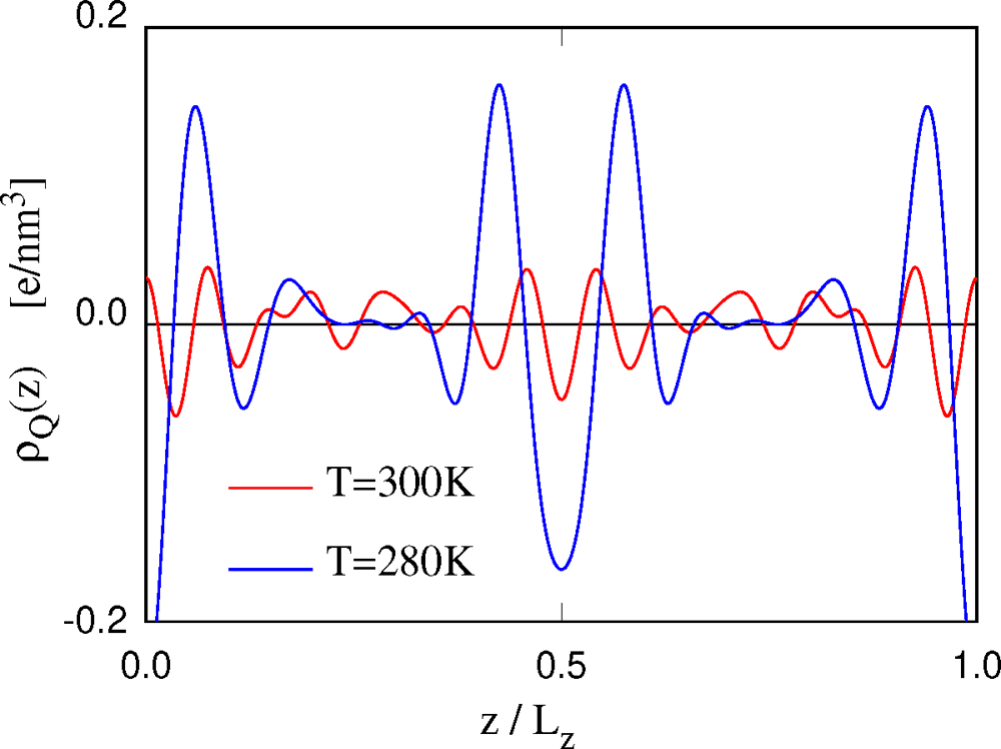
Charge density profile for the POPC+60 [DxC10][Cl]_2_/water along the direction *z* perpendicular to the bilayers.

Charge density profiles suffer from the same limitations of the number or mass density profiles in dealing with rippled bilayers. Their computations, however, suggest an apparent reason for rippling, as shown in Figure 11, comparing the charge density profile for sample IV at *T* = 280 K (fluid planar phase) and for the same sample IV at *T* = 300 K (ripple phase). Rippling, in particular, greatly decreases the amplitude of the charge density oscillations ρ_*Q*_(*z*), well beyond the expected effect of increasing *T* by 20 K (that have been estimated by comparing the charge density profile of sample IV at *T* = 300 and *T* = 320 K). However, ρ_*Q*_(*z*) directly determines ρ̃_*Q*_(*q*_*x*_ = 0;*q*_*y*_ = 0;*q*_*z*_), whose contribution to the electrostatic Hartree energy ∝ρ̃_*Q*_(**q**)ρ̃_Q_^*^(**q**)/|*q*_*x*_^2^ + *q*_*y*_^2^ + *q*_*z*_^2^| is sizable because of the vanishing of *q*_*x*_ and *q*_*y*_. Therefore, the reduction in the amplitude of ρ_*Q*_(*z*) could indeed represent the driving force for rippling, or, at least, it contributes to it.

**Figure 12:**
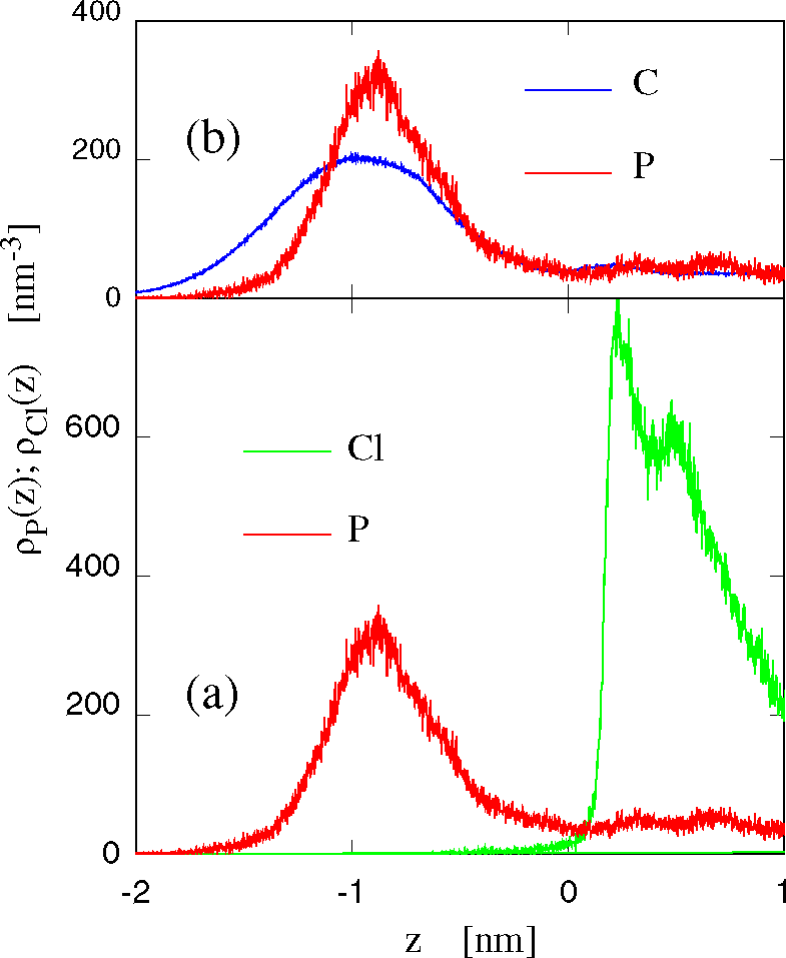
Number density profiles with respect to the local position of the interface. Negative and positive *z* correspond to the lipid and water side of the interface, respectively. (a) P belonging to [DxC10]^2+^ and [Cl]^−^ in sample IV at *T* = 300 K. (b) Number density profile of P and C belonging to [DxC10]^2+^ computed again for sample IV. The C profile has been scaled down in such a way that the P and C distributions cover the same area.

A more informative view of the interfacial structure is provided by the intrinsic density profiles computed with respect to the instantaneous surface separating lipids from the other components. These profiles are reported in Figure 12. Part a compares the density distribution of the P-center of phosphonium with the distribution of [Cl]^−^ anions at the interface, showing a clear separation of charge, giving origin to a strong electrostatic double layer. Part b compares the density profile of carbon and phosphorus atoms belonging to the [DxC10]^2+^ cation. The two distributions have been scaled in order to cover the same area. The comparison shows that the C atoms belonging to the diphosphonium alkyl tails sit deeper than the cationic-P in the bilayer, apparently because of their known affinity for the hydrocarbon tails of POPC. These configurations, with P from the cation absorbed close to the polar head of POPC and the alkyl chains entering the lipid hydrophobic range, appear to be very stable and to be difficult to reverse in order for the cation(s) to jump onto the adjoining leaflet. The intrinsic density profiles for the pure POPC/water sample (sample I) confirm that, as expected, water and lipids mix in the narrow layer occupied primarily by the polar (zwitterionic) heads of POPC, whose width, in all samples, is about 0.8 nm. Water virtually does not penetrate beyond this range toward the hydrophobic domain of the hydrocarbon tails of POPC. This strict separation of water and hydrophobic lipid tails is retained in all samples we simulated, as shown by the intrinsic density profiles reported in Figure S6 of the Supporting Information. In other terms, water does not follow the cations below the polar range of the lipid bilayer. Therefore, up to the ionic concentrations considered in our simulations, no swelling of the lipid bilayers due to water is observed. Moreover, it is conceivable to think that the free energy activation required for the absorption of cations below the water/lipid interface is due primarily to the dehydration of the cation.

The volume of each lipid bilayer in the simulated samples has been determined by the two methods mentioned in Section II, i.e., integration over the bilayer plane of the width using a regular mesh or a random distribution of the same number of points, equivalent to Monte Carlo integration. The two sets of results are equivalent well within the error bar. Table 3 reports the data from the regular mesh computation, since these are more directly reproducible. Although the determination of the two interfaces limiting each bilayer is based on the coordinates of the lipids only, the computed volume *V* accounts also for the volume of the cations absorbed between the interfaces, as reflected in the data of Table 3. First of all, the volumes of all salt-added systems are larger than those of the pure POPC/water sample I. Moreover, the extra volume with respect to sample I shows, as expected, a nearly linear dependence on the number of cationic groups added to the sample. The *δV* parameter reported in Table 3 represents the standard deviation of volumes over the set of configurations sampled by simulation over time. In many ways, this parameter measures the compressibility, i.e., the softness of the bilayer, which increases with increasing salt concentration, underlining the progressive but still limited destabilization due to the absorption of cations into the lipid phase. The relation of *δV* and compressibility, however, is not a simple proportionality because of the coupling of the lipid bilayer with the hydration layer.

**Table 3:** Volume of a Lipid Bilayer in the Simulated Samples^a^

label	*T* [K]	phase	⟨*h*⟩ [nm]	⟨*δh*⟩ [nm]	*V* = ⟨*hA*_0_⟩ [nm^3^]
I	280	P	4.68	1.2	943.9
	300	P	4.69	0.95	952.4
	320	P	4.71	1.1	964.0
IV	280	P	4.76	0.9	956.2
	300	R	4.88	1.1	989.3
	320	P	4.93	1.1	1009.2
VII	300	R	4.89	1.1	997.0
VIII	300	R	5.09	1.2	1035.3

a Each sample contains two lipid bilayers, each consisting of 680 POPC molecules arranged on two leaflets. The *δV* parameter measures the standard deviation in the volume of each bilayer averaged over time, and it is closely related to the bilayer volume compressibility (see text). The error bar is implicitly given by the number of digits.

Surface tension and bending rigidity are important thermomechanical properties of membranes and lipid bilayers in particular, measuring the free energy required to increase the surface area and the curvature, respectively. Their variation upon the addition of ILs is well documented,^23,57,58^ and it is often associated with cytotoxic effects of ILs. While the application of eq 7 to planar bilayers is simple,^25,26^ the application of the modified eq 8 to a ripple phase is less straightforward. As an example, let us consider POPC+60 [DxC10][Cl]_2_ at *T* = 300 K, whose ripple state is apparent from the wave-like profile of the bilayer in the (*xz*) plane (see Figure 7a). The lipid bilayer, however, is fluid, and the ripple, although slowly, moves randomly in time. As a consequence, the long-term averages *C̅*_**q**_ in eq 8 vanish even in the ripple phase, and the ⟨(*C*_**q**_ – *C̅*_**q**_)(*C*_–**q**_ – *C̅*_–**q**_)⟩ loses its meaning of square fluctuation around the ripple equilibrium shape. The correct application of eq 8, therefore, requires averaging ⟨(*C*_**q**_ – *C̅*_**q**_)(*C*_–**q**_ – *C̅*_–**q**_)⟩ over times that are long with respect to the frequency of short wavelength fluctuations and short with respect to the thermal diffusion of the ripple phase.

**Figure 13:**
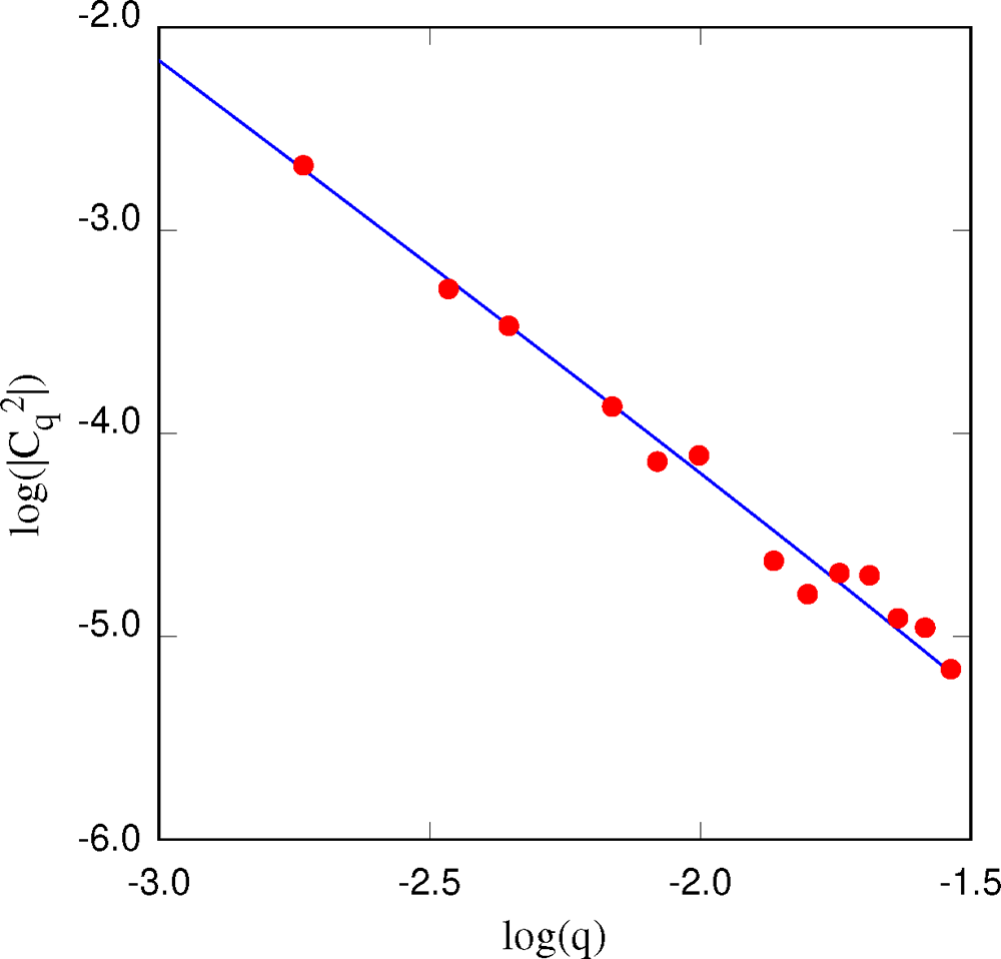
Fit (blue line) of the long wavelength range of ⟨|*C*_*q*_|^2^⟩ by eq 7. The average of |*C*_*q*_(*t*)|^2^ (red dots) has been carried out on 100 ns. The data refer to the POPC/water+60 [DxC10][Cl]_2_ sample at *T* = 300 K.

We verified by testing that a suitable time window for averaging ⟨(*C*_**q**_ – *C̅*_**q**_)(*C*_–**q**_ – *C̅*_–**q**_)⟩ is 100 ns, since with this choice the results are not very sensitive to slight variations of this interval. Therefore, we divided the last 1 μs simulation of each sample into 10 subintervals. Again for each sample, we applied eq 8 to the average computed on each interval, obtaining 10 values of γ and *K*_*c*_, whose average and estimated error are listed in Table 1. The fit of ⟨(*C*_**q**_ – *C̅*_**q**_)(*C*_–**q**_ – *C̅*_–**q**_)⟩ by eq 8 is carried out on the −3 ≤ log (*q*) ≤ – 1.5 Å ^–1^ range to approach the long wavelength limit at the same time excluding a few longest wavelength contributions that are likely to be affected by periodic boundary conditions. An example of fit on a single time window is shown in Figure 13. For all samples, this procedure provides a fairly well-defined value of γ, while, as expected, *K*_*c*_ is affected by significantly larger uncertainties.

The data collected in Table 1 show that, for the clean POPC sample, γ and *K*_*c*_ agree with those of previous computations^25,26^ to within the error bar. At *T* = 300 K, addition of electrolytes at concentration sufficiently low to retain the bilayer planarity (sample VI) increases slightly the surface tension, and decreases the bending rigidity. At high electrolyte concentration, with the bilayer in the ripple phase (samples IV, VII and VIII at *T* = 300 K), the surface tension is higher, and the bending rigidity is somewhat lower than in the pure POPC case.

Decreasing the temperature to *T* = 280 K restores the planarity of the bilayer even with electrolyte, increasing both γ and *K*_*c*_. As expected, the opposite effect is observed when temperature is increased to *T* = 320 K.

The (self-)diffusion of lipids and mono- and dication species has been characterized following the (irreversible) absorption of the cations into the POPC range, and, to first approximation, represents a nearly 2D random walk of all these species along the bilayer. For all samples, the last 600 ns of the MD trajectory have been considered for the computation of the self-diffusion coefficient. In samples containing either [DxC10][Cl]_2_ or [P_6,6,6,6_][Cl], this time interval might slightly overlap with the final stages of cation equilibration within the lipid phase, but subaverages over shorter time intervals show that the drift of the diffusion coefficient over the last 600 ns of MD is much less than the uncertainty due to time averaging. On the other hand, the 600 ns long averaging time provides very smooth data for the mean square displacement as a function of time for molecules as big as POPC or [DxC10]^2+^ (see Figure S7 in the Supporting Information), thus allowing a robust estimate of diffusion coefficients as small as ∼1 × 10^–8^ cm^2^/s. The self-diffusion coefficient of POPC in the neat lipid/water sample has been estimated at *D*_*POPC*_ = 2.4 ± 0.1 × 10^–8^ cm^2^/s. This value agrees with previous estimates by our group,^25,26^ and it is compatible with the computational results of ref (27), while it disagrees with those of ref (59), obtained, however, under somewhat different conditions. The results of experimental measurements are rather scattered, with discrepancies reflecting primarily the different time scale of different measurement approaches. Nevertheless, the pulsed-field gradient NMR (PFG-NMR) value (*D* = 1.8 ± 0.9 × 10^–8^ cm^2^/s) of ref (60) agrees well with the present result, which agrees even better with the FRAP estimate (*D* = 2.5 ± 0.9 × 10^–8^ cm^2^/s) of the same ref (60). In this respect, it might be useful to consider that PFG-NMR and molecular dynamics both measure self-diffusion, while FRAP probes directly Fick’s diffusion, whose conversion to self-diffusion, requiring a detailed knowledge of the system thermodynamic functions, is not mentioned in ref (60). Therefore, the excellent agreement of the computed and FRAP diffusion coefficients is likely to be partly fortuitous.

The computed diffusion coefficients that are relevant for our discussion are collected in Table 4. Since [DxC10][Cl]_2_ and [P_6,6,6,6_][Cl] of samples IV and VII are added in the w1 water interlayer only and enter the adjacent (B and C) leaflets without diffusing into the farthest (A and D) leaflets, the diffusion coefficient has been computed independently for lipids belonging to the B, C (contaminated) and to the A, D (uncontaminated) leaflets. For these systems, the diffusion coefficient of POPC in leaflets B, C is denoted by *D*_*POPC*–*In*_, while *D*_*POPC*–*Out*_ denotes the diffusion constant for leaflets A and D. No distinction is made between in and out for samples I and VIII. Of course, in all cases, the distinction between the two sets of leaflets is relevant primarily on microscopic time scales, because on macroscopic time scales the (apparently slow) exchange of cations among leaflets will certainly reduce the difference (but not fully equalize, since the bilayer is impermeable to [Cl]^−^) of composition among all leaflets.

**Table 4:** Self-Diffusion Coefficient of POPC and Cations Computed from the Einstein Formula in the *xy* Plane^a^

sample I	sample IV	sample VII	sample VIII
*D*_*POPC*_ = (2.4 ± 0.1) × 10^–8^	*D*_[*DxC*10]^2+^_ = (1.1 ± 0.1) × 10^–8^	*D*_[*P*_6,6,6,6_]^+^_ = (7.4 ± 1) × 10^–9^	*D*_[*P*_6,6,6,6_]^+^_ = (1.2 ± 0.1) × 10^–8^
	*D*_*POPC*–*In*_ = 1.2 ± 0.1 × 10^–8^	*D*_*POPC*–*In*_ = 8.8 ± 1 × 10^–9^	*D*_*POPC*–*In*_ = 1.2 ± 0.1 × 10^–8^
	*D*_*POPC*–*Out*_ = 2.15 ± 0.1 × 10^–8^	*D*_*POPC*–*Out*_ = 2.1 ± 0.1 × 10^–8^	*D*_*POPC*–*Out*_ = 1.25 ± 0.1 × 10^–8^

a Temperature of all samples is *T* = 300 K, and diffusion coefficients are measured in cm^2^/s.

The first message apparent from the data is that in all salt-containing samples, diffusion is very similar for the cations and for POPC in the same leaflet. Moreover, *D*_*POPC*–*In*_ is significantly lower than the POPC diffusion coefficient in the neat POPC/water sample I. Both features point to a strong coupling in the dynamics of these two species, emphasizing their thermodynamic affinity. Despite its mass advantage, [P_6,6,6,6_]^+^ diffuses more slowly than [DxC10]^2+^ once they are absorbed in the lipid bilayer, while the reverse is true in water (see the data for samples IV and VII in Table 4). Low mobility might result from a stronger or more complex interaction with POPC in the case of [P_6,6,6,6_]^+^ compared to [DxC10]^2+^. Again for samples IV and VII, POPC diffusion in leaflets A and D (*D*_*POPC*–*Out*_) is nearly unaffected by the absorption of cations in the adjoining leaflet. Perhaps not surprisingly, POPC diffusion in all leaflets of sample VIII is about the average of *D*_*POPC*–*In*_ and *D*_*POPC*–*Out*_ in sample VII, and drives a slight recovery of the diffusion coefficient of [P_6,6,6,6_]^+^ in the same system.

Lipid–lipid radial distribution functions, computed assuming a planar 2D structure show limited variations among the different samples, including those samples that display rippling (see Figure S8 in the Supporting Information). No clustering of cations in the lipid bilayers is expected because of their mutual Coulombic repulsion, and no clustering is apparent in the in-plane radial distribution functions.

## IV Summary and Discussion

The effect of two alkylphosphonium chloride salts on the structure and stability of the hydrated POPC phospholipid bilayer has been investigated by MD simulations based on an empirical (near-)atomistic force field. The two phosphonium chlorides, based on the [DxC10]^2+^ dication and [P_6,6,6,6_]^+^ cation, display a marked biological activity, both being toxic to bacteria, and [DxC10][Cl]_2_ but not [P_6,6,6,6_][Cl] being innocuous to eukaryotic cells. Simulations were aimed at investigating the microscopic mechanism by which they interact and possibly disrupt the lipid fraction of biomembranes, eventually contributing to shed light on their different effect on cells. Needless to say, the single-lipid (POPC) lipid bilayer is far too simple to provide direct information on biological activity, and the focus of our discussion is on the understanding of IL-phospholipid interactions and on the general insight one can gain from computations on the effect of ILs on lipid bilayers, in support of biology but also nanoscience applications.

The results show that the effect of the mono- and dication phosphonium salts is remarkably similar in several aspects. First, both cationic species are readily absorbed by the lipid bilayer, showing a water–lipid partitioning largely skewed toward the latter phase. At all concentrations we simulated, the highly hydrophilic [Cl]^−^ anion remains in the water interlayer between the POPC bilayers, resulting in a sizable charge separation at the interface. Because of the low affinity for alkylphosphonium, water does not follow the cations into the bilayer. Perhaps also because of this fact, up to the (high) concentration of 1 |*e*| charge per 11 POPC molecules, the bilayers retain their integrity and impermeability. The most apparent structural effect of the salts at medium-high concentration is the formation of a stationary, wide amplitude ripple at (*P*, *T*) conditions at which the hydrated POPC bilayer is stable in the planar fluid phase. Analysis of the density and charge density profiles across the bilayers suggests that rippling might be driven by the increasing Hartree energy of the planar electrostatic double layer with increasing absorption of cations into the lipid phase.

Besides these qualitative similarities, quantitative differences in the effects of [DxC10][Cl]_2_ and [P_6,6,6,6_][Cl] are also apparent. The first difference is in the short-time absorption kinetics (up to ∼1 μs), that, as expected, is faster for [P_6,6,6,6_]^+^ than for [DxC10]^2+^. The ratio of the two time scales can be estimated a priori, considering the different diffusion coefficients of the two species. This observation emphasizes the role of diffusion in the absorption process. On a slightly longer time scale (∼1 μs), the absorption kinetics of [DxC10]^2+^ undergoes a transition to a somewhat slower mode, whose explanation could be based on the following observation. In a first *t* ∼ 1 μs stage of absorption, each dication tends to insert one phosphonium headgroup only into the bilayer, while the other remains in solution, stretching the −C10– linker. Only in a second stage (*t* > 1 μs), also the second phosphonium headgroup enters the lipid range, but this second process has to overcome a slightly higher barrier. Eventually, also in the [DxC10][Cl]_2_ case, nearly all dications become absorbed below the water/lipid interface. The reason for the higher barrier opposing the absorption of the second headgroup is that, because of the −(CH_2_)_10_– linker, this second cationic moiety has to enter the bilayer at short distance from the first, possibly finding a stiffer lipid conformation perturbed by the presence of the first headgroup. In all samples and for both cation choices, the activation free energy for absorption might be partly due to the dehydration of cations in entering the lipid bilayer.

While diffusion, as expected, affects absorption, also the reverse is true, i.e., absorption affects diffusion of all species. Comparison of POPC diffusion for different salt concentrations and mode of insertion (into w1 only or in both w1 and w2) shows that absorptions of cations decreases the mobility of POPC, and at equal cationic charge concentration, the effect is stronger for [DxC10]^2+^ than for [P_6,6,6,6_]^+^. Addition of cations in one leaflet only affects slightly the POPC diffusion in the adjacent leaflet, although some coupling of the lipid diffusion in the two leaflets is observed.

In all cases, the area ⟨*A*⟩ of the lipid/water interface is much wider than its projection ⟨*A*_0_⟩ on the (*xy*) plane ([⟨*A*⟩ – ⟨*A*_0_⟩]/⟨*A*_0_⟩ > 70%). Rippling increases even further and significantly this difference, reaching up to 90%. Fluctuations around the average planar or rippled surface allow one to estimate the two basic thermodynamic properties of the interface, i.e., the interfacial tension γ and the bending rigidity *K*_*c*_. It is found that the interfacial tension γ increases upon adding the phosphonium salts, either [DxC10][Cl]_2_ or [P_6,6,6,6_][Cl]. The bending rigidity *K*_*c*_, instead, seems to have a nonmonotonic dependence on the interfacial charge density due to the absorption of cations. A picture consistent with but not strictly proven by the available computational data suggests that, at first, absorption of cations (i.e, going from sample I to sample III, at *T* = 300 K) decreases significantly *K*_*c*_, causing the transition to the ripple phase. The transition, in turn, restores part of the bending rigidity (samples IV and VII), which increases again, although slightly, with increasing charge density and with adding cations to both leaflets of the bilayer (sample VIII). The validity of this interpretation depends also on the fact that *K*_*c*_ has to nearly vanish at the ripple transition, taking place with increasing cation concentration between sample III and sample IV for [DxC10][Cl]_2_ and between sample VI and sample VII for [P_6,6,6,6_][Cl]. We emphasize that this picture is tentative since the determination of *K*_*c*_ from fluctuations is rather uncertain (more uncertain than the γ determination), and several more samples of different salt concentration are needed to unambiguously determine the dependence of *K*_*c*_ on the interfacial charge density. Nevertheless the proposed sketch provides a direction for further computational research.

It is tempting to suggest that the changes from planar to ripple phase and the corresponding changes of γ and *K*_*c*_ could contribute to the biological effects of alkylphosphonium salts. Admittedly, the differences in the properties of POPC bilayers resulting from the addition of [DxC10][Cl]_2_ or [P_6,6,6,6_][Cl] do not seem to be sufficient to explain the differences in toxicity of the two compounds, [DxC10][Cl]_2_ being lethal to bacteria and innocuous to eukaryotic cells^6^ and [P_6,6,6,6_][Cl] being toxic to both. As already stated, bilayers made of the single POPC phospholipid molecule are far too simple to provide a reliable picture of toxicity, as this does not account even for the composition difference in the biomembrane of bacteria, having a significant anionic signature, and eukaryotic cells, made predominantly by zwitterionic lipids such as POPC. However, our investigation might contribute to research in this context, which has obvious implications for pharmacology and drug delivery in particular. If the two-stage absorption kinetics has a role in determining the biological effect of different phosphonium species, it could be amplified or reduced by using asymmetric dications instead of geminals.

An interesting question that might be addressed by future simulations concerns the possible protective effect of cholesterol on biomembranes, which could limit the changes and disruptions due to the absorption of phosphonium cation species, analogous to the observations of ref (61). Moreover, the affinity of lipids for cationic (and phosphonium in particular) head groups might provide the opportunity to link together bilayers or to tether lipid micelles and vesicles to surfaces or among themselves, using di- or multiphosphonium species joined by suitable chains. These approaches could provide a stronger link than currently achievable by polymers terminated by cholesterol head-groups.^62^ Tethering and linking lipid nanostructures could also result in new materials, possibly useful for nanofabrication, rheology, and tribology.

## Supporting Information Available

Topographic view of a lipid–water interface, a snapshot of bilayers in water displaying poration resulting from the sudden addition of salt at high concentration, plots showing the time dependence of the [DxC10]^2+^ absorption at low concentration, water and lipid density profiles computed with respect to the instantaneous surface identified as the lipid/water interface, the mean square displacement of POPC and [DxC10]^2+^ following the absorption of the dications into the lipid phase, and the in-plane (2D) radial distribution functions of lipids and P^+^ atoms belonging to [DxC10]^2+^ in a system with high salt concentration and bilayer in the ripple phase (PDF)
Input data required by Gromacs to simulate sample IV and sample VII (ZIP)
