# Supplementary material for: Absorption of Phosphonium Cations and Dications into a Hydrated POPC Phospholipid Bilayer: A Computational Study
Source: J Phys Chem B. 2022 Jun 6;126(23):4272–88. doi: 10.1021/acs.jpcb.2c02212 (PMC9207913; doi:10.1021/acs.jpcb.2c02212)
Supplement: Supplementary file 1 — jp2c02212_si_001.pdf [file jp2c02212_si_001.pdf]

The Absorption of Phosphonium Cations and Dications into a Hydrated POPC Phospholipid Bilayer: a Computational Study

V. V. S. Pillai,[(1,2)] P. Kumari,[(1,2)] A. Benedetto,[(1,2,3,*)] D. Gobbo,[(4,5)] and P. Ballone,[(1,2)]

(1) School of Physics, University College, Dublin, Dublin 4, Ireland

(2) Conway Institute for Biomolecular and Biomedical Research, University College, Dublin, Dublin 4, Ireland

(3) Department of Sciences, University of Roma Tre, I-00154 Rome, Italy

(4) School of Pharmaceutical Sciences and ISPSO, University of Geneva, Rue Michel-Servet 1, CH-1211, Geneva 4, Switzerland and

(5) Computational and Chemical Biology, Fondazione Istituto Italiano di Tecnologia, I-16163 Genova, Italy

Corresponding Author: A. Benedetto Antonio.Benedetto@ucd.ie

I. AFM IMAGE OF THE WATER/LIPID INTERFACE

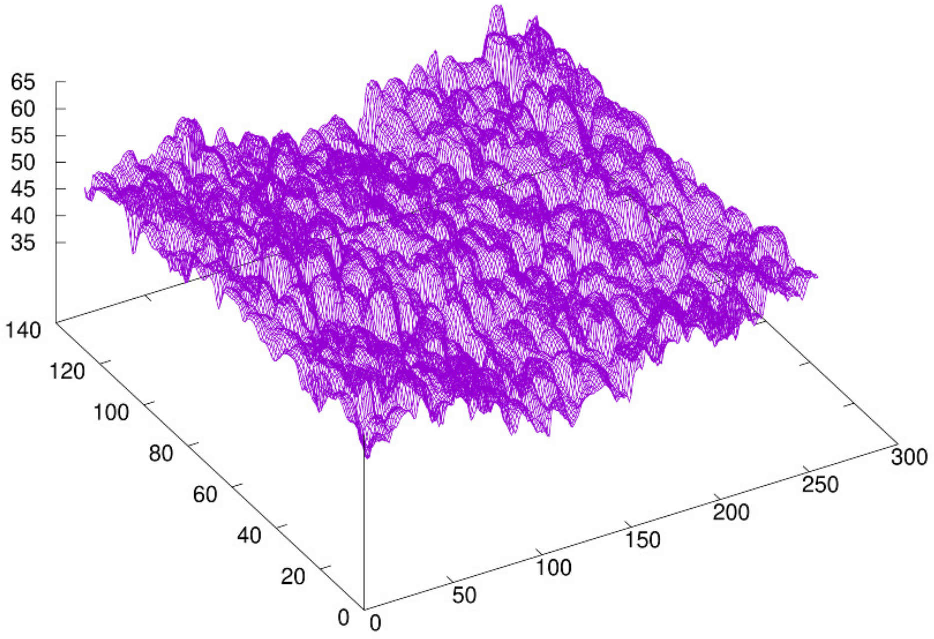

FIG. S1: Geometric surface marking the instantaneous position of the interface between water and lipids determined by the virtual AFM approach described in the text. Coordinates are given in Å .

II. EFFECT OF THE SUDDEN INSERTION OF [DXC10][CL]$_2$ AT HIGH CONCENTRATION

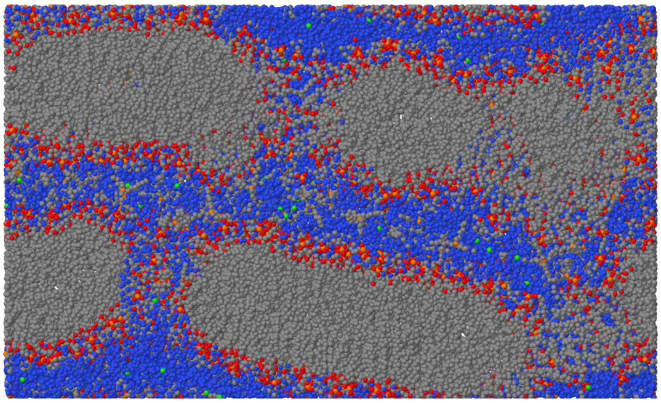

FIG. S2: Hydrated POPC bilayers disrupted by the sudden insertion of 60 [DxC10][Cl]$_2$ ions into the w1 water interlayer. The color code is as follows. Carbon: black; oxygen in POPC: red; water oxygen: blue; sulphur: yellow; phosphorus: orange; the nitrogen in POPC is also blue. The strong sudden perturbation created water pores across the bilayers.

III. APPROACH OF A DICATION TO THE LIPID SURFACE

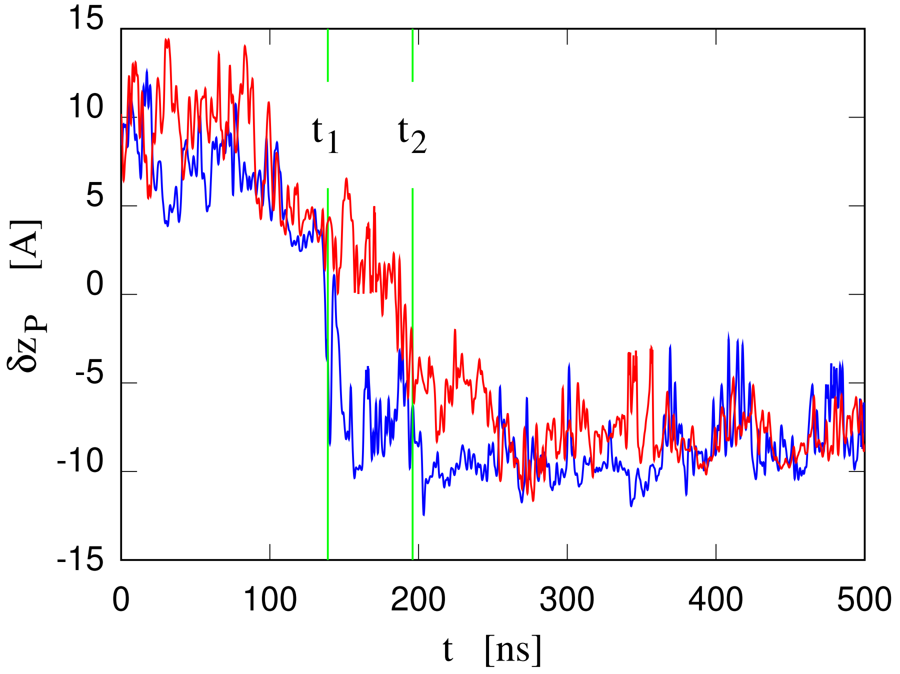

FIG. S3: Distance of the P atoms of $[DxC10]^{2+}$ above the water / lipid interface under which they are eventually absorbed. t_1 and t_2 mark the times at which the first and the second P atom enter the surface, respectively. Data from the simulation of Sample II, consisting of POPC / water + 2 $[DxC10][Cl]_2$. The plot for the other cation is qualitatively equivalent. Qualitatively similar results are obtained also for Sample V, consisting of POPC, water and 4 $[P_{6,6,6,6}][Cl]$ ion pairs.

IV. APPROACH OF A DICATION TO THE LIPID SURFACE

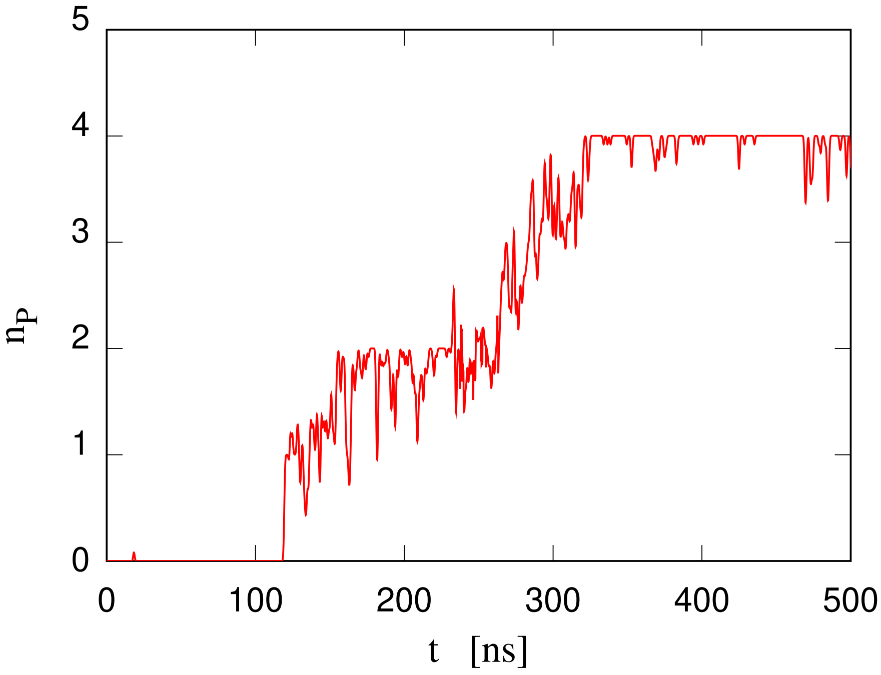

FIG. S4: Number of P atoms belonging to $[DxC10]^{2+}$ absorbed under the lipid surface as a function of time following the insertion of two dications in the w1 water interlayer of the neat POPC / water sample. The plot is the sum of contribution from the two cations and for the two lipid / water interfaces above and below w1. Data have been slightly smoothed to avoid sharp steps.

V. NUMBER OF CL^- ANIONS ABSORBED UNDER THE WATER / LIPID INTERFACE IN SYSTEM IV

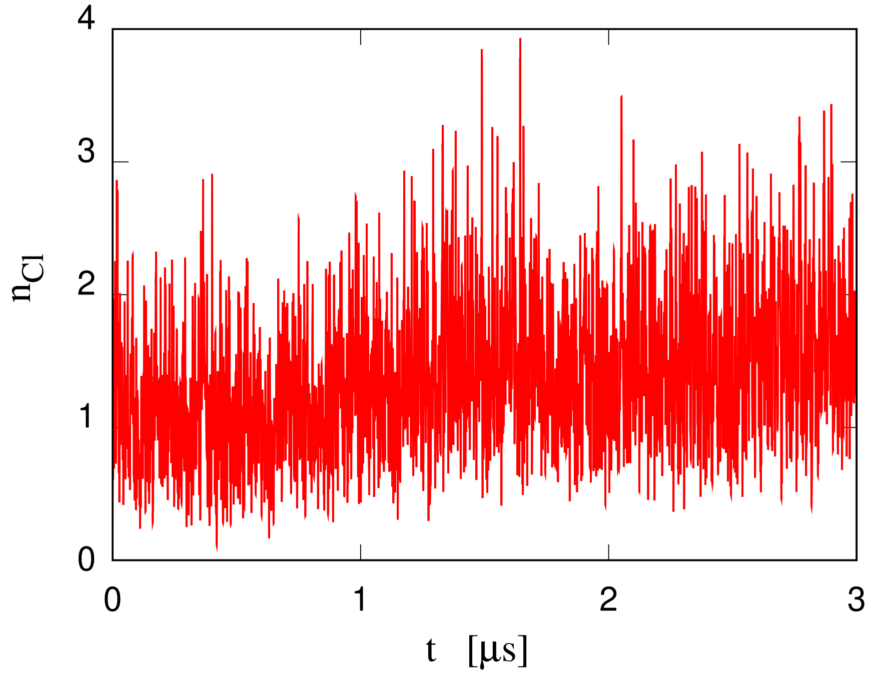

FIG. S5: Number of Cl^- anions absorbed under the water / lipid interface as a function of time. Data from the simulation of System IV, consisting of POPC / water + 60 $[DxC10][Cl]_2$.

VI. DENSITY DISTRIBUTION WITH RESPECT TO THE INSTANTANEOUS LIPID / WATER INTERFACE

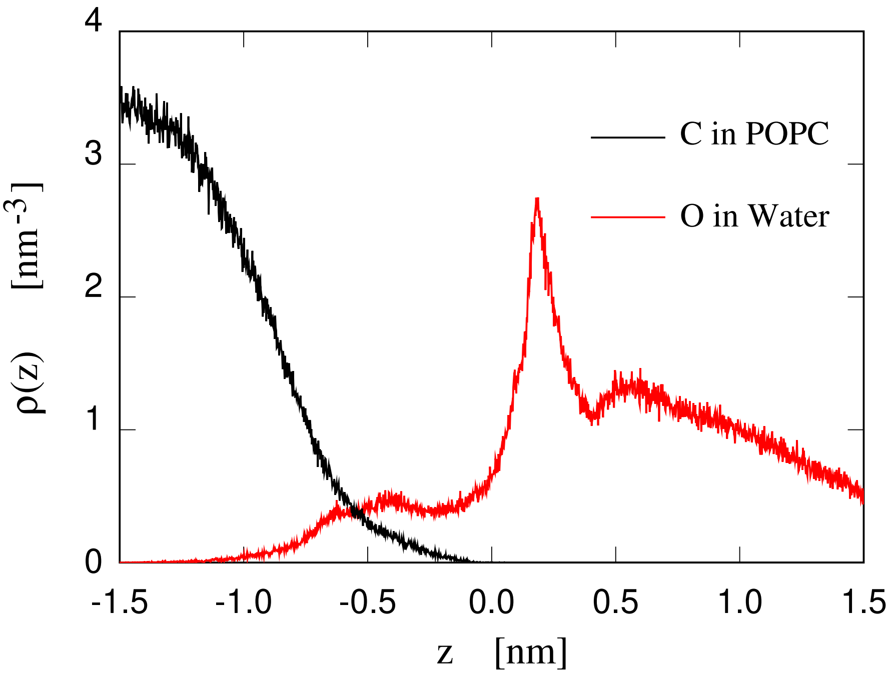

FIG. S6: Density distribution of C $\in$ POPC and O $\in$ H_2O computed with respect to the instantaneous lipid / water interface, computed for Sample IV, consisting of POPC / water + 60 [DxC10][Cl]$_2$. The two curves illustrate the separation of water and lipids, whose limited mixing occurs only at the polar head of POPC, even for systems in the ripple phase and high salt concentration.

VII. MEAN SQUARE DISPLACEMENT OF POPC AND CATIONS IN SYSTEM IV

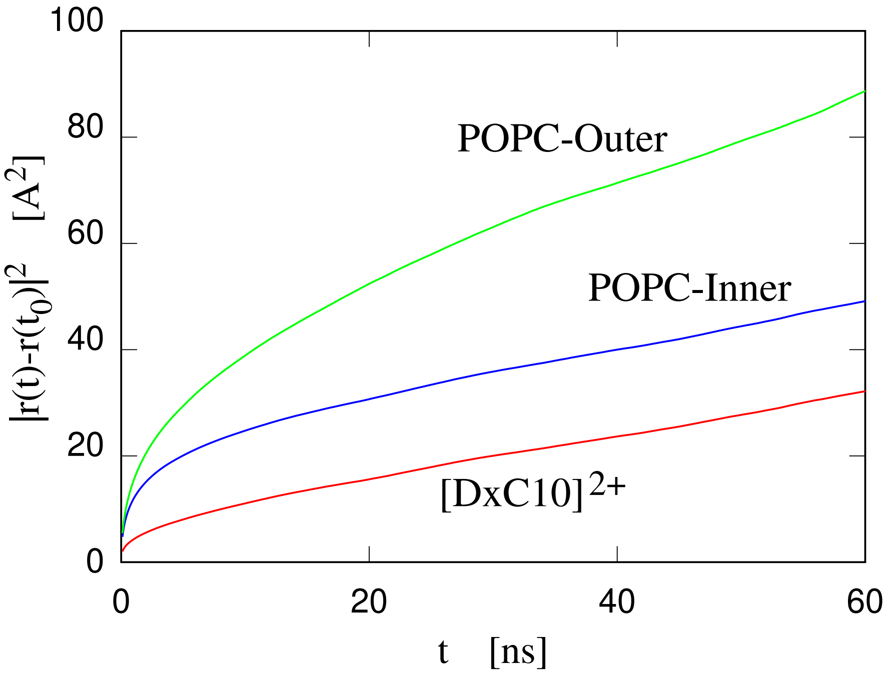

FIG. S7: Mean square displacement (per atom) of POPC and $[DxC10]^{2+}$ in System IV, consisting of POPC / water + 60 $[DxC10][Cl]_2$. POPC-Inner refers to POPC molecules in leaflets B and C, adjacent to the w1 water interlayer in which $[DxC10][Cl]_2$ has been inserted. POPC-Outer refers to POPC molecules in leaflets A and D, adjacent to the w2 water interlayer free of $[DxC10][Cl]_2$ ion pairs. The square displacement is averaged over the initial time t_0 considering the last 600 ns of the simulation lasting 3.488 μs. The diffusion constants have been determined using the linear fit of the mean square displacement for $40 \leq t \leq 60$ ns.

VIII. IN-PLANE (2D) RADIAL DISTRIBUTION FUNCTIONS

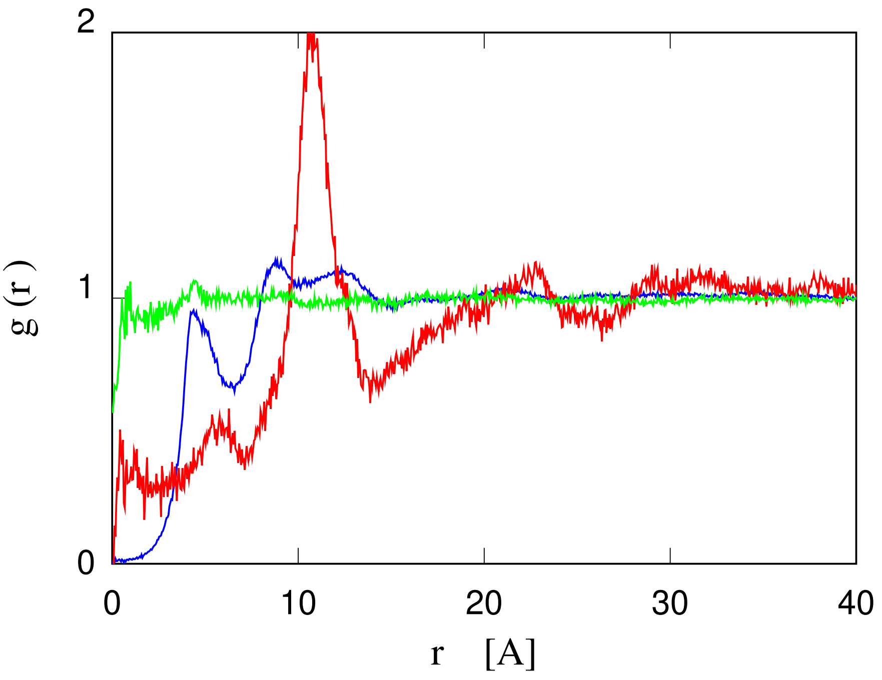

FIG. S8: 2D radial distribution functions for species belonging to or absorbed into the same lipid leaflet. Red line: P^+-P^+, with P^+ belonging to $[DxC10]^{2+}$; blue line: POPC-POPC; green line: POPC-P^+. Data for Sample IV, consisting of POPC / water + 60 $[DxC10][Cl]_2$. The non-vanishing values at $r = 0$ and the lack of POPC-cation radial correlation are due to the fact that separations are computed in 2D, while the 3D distance, i.e., including the separation along z, is always non negligible. The 2D radial distribution functions confirm that cations do not show a tendency to cluster into the lipid phase.
